# Calibration and application of the Chemcatcher® passive sampler for monitoring acidic herbicides in the River Exe, UK catchment

**DOI:** 10.1007/s11356-018-2556-3

**Published:** 2018-06-25

**Authors:** Ian Townsend, Lewis Jones, Martin Broom, Anthony Gravell, Melanie Schumacher, Gary R. Fones, Richard Greenwood, Graham A. Mills

**Affiliations:** 1 South West Water Ltd, Peninsula House, Rydon Lane, Exeter, Devon EX2 7HR UK; 2 Natural Resources Wales, NRW Analytical Services at Swansea University, Faraday Building, Swansea University, Singleton Campus, Swansea, SA2 8PP UK; 3 School of Earth and Environmental Sciences, University of Portsmouth, Burnaby Road, Portsmouth, PO1 3QL UK; 4 School of Biological Sciences, University of Portsmouth, King Henry I Street, Portsmouth, Hampshire PO1 2DY UK; 5 School of Pharmacy and Biomedical Sciences, University of Portsmouth, White Swan Road, Portsmouth, Hampshire PO1 2DT UK

**Keywords:** Acidic herbicides, Passive sampling, Chemcatcher^®^, Calibration, Field trials, River catchments, Water quality monitoring

## Abstract

Acidic herbicides are used to control broad-leaved weeds. They are stable, water-soluble, and with low binding to soil are found frequently in surface waters, often at concentrations above the EU Drinking Water Directive limit of 0.10 μg L^−1^. This presents a problem when such waters are abstracted for potable supplies. Understanding their sources, transport and fate in river catchments is important. We developed a new Chemcatcher^®^ passive sampler, comprising a 3M Empore™ anion-exchange disk overlaid with a polyethersulphone membrane, for monitoring acidic herbicides (2,4-D, dicamba, dichlorprop, fluroxypyr, MCPA, MCPB, mecoprop, tricolpyr). Sampler uptake rates (*R*_*s*_ = 0.044–0.113 L day^−1^) were measured in the laboratory. Two field trials using the Chemcatcher^®^ were undertaken in the River Exe catchment, UK. Time-weighted average (TWA) concentrations of the herbicides obtained using the Chemcatcher^®^ were compared with concentrations measured in spot samples of water. The two techniques gave complimentary monitoring data, with the samplers being able to measure stochastic inputs of MCPA and mecoprop occurring in field trial 1. Chemcatcher^®^ detected a large input of MCPA not found by spot sampling during field trial 2. Devices also detected other pesticides and pharmaceuticals with acidic properties. Information obtained using the Chemcatcher^®^ can be used to develop improved risk assessments and catchment management plans and to assess the effectiveness of any mitigation and remediation strategies.

**Electronic supplementary material:**

The online version of this article (10.1007/s11356-018-2556-3) contains supplementary material, which is available to authorized users.

## Introduction

Auxin mimicking acidic herbicides, including phenoxy- and pyridyloxy-acids, are applied widely to control broad-leaved weeds in grassland and some cereal crops and to combat unwanted woody plants in forests and railways (HSE 2018). Many of these compounds exhibit high aqueous solubility, are stable under typical environmental conditions and show little tendency to bind to soil (PPDB 2018). Consequently run-off into surface waters is facile whilst widespread usage increases the likelihood of point source pollution arising from inappropriate application practices or poor husbandry in terms of storage conditions, machinery wash down or the disposal of excess material. Prevailing climatic conditions, geography and geology have resulted in the agriculture of the far South West of England being dominated by grassland used for cattle farming with large attendant acreages of both permanent and temporary grassland (Fig. S1). Associated weed control for these areas involves heavy usage of pesticides including acidic herbicides such as clopyralid, fluroxypyr, MCPA, mecoprop and triclopyr (Table S1), primarily in spring and early summer, although significant applications can occur later in the year.

South West Water Ltd. (SWW) is responsible for the provision of drinking water in the far South West of England (counties of Cornwall, Devon and parts of Dorset and Somerset). The company uses an extensive water quality monitoring programme that targets a wide range of pesticides (60 compounds, including 16 acidic herbicides). Acidic herbicides are detected regularly in raw surface waters in a number of major South West river catchments (e.g. Exe, Fowey and Tamar). Frequently, their concentrations exceed the European Union’s Drinking Water Directive limit of 0.1 μg L^−1^ for any pesticide (referred to within the UK water industry as the prescribed concentration value (PCV) which is legally binding) (EU Directive 1998). These rivers are strategically important for the provision of surface-derived drinking water supplies for the region. In order to ensure consistent regulatory water quality compliance, some surface waters are treated at the supply works by contact with granular activated carbon in an attempt to remove these pollutants. This removal process is costly and has a high-energy footprint, since there is a requirement for periodic regeneration of the carbon in order to maintain good removal efficiency. Some heavily used, highly polar and water-soluble pesticides, including the acidic herbicide, clopyralid, and the molluscicide, metaldehyde, are not efficiently removed by granular activated carbon, and this, therefore, poses a continued threat to drinking water quality (Castle et al. 2017). Controlling the input of these pollutants via well-targeted catchment management initiatives is therefore essential.

SWW, together with Westcountry Rivers Trust and the Wildlife Trusts of Devon and Cornwall, have recently implemented an initiative called ‘Upstream Thinking’ (SWW 2017). This novel environmental scheme aims at improving the quality of raw surface waters in key catchments in the South West of England, whilst enhancing their ecological status in accordance with European Union’s Water Framework Directive (EU Directive 2000). It is one of the first environmental improvement programmes in the UK to look at all the issues influencing water quality and quantity across entire catchments. The scheme aims to reduce inputs of sediment, cattle slurry, silage liquor and pollutants, such as nitrate and pesticides, into watercourses. Identifying tributaries that are the primary source of pesticide pollution and assessing the effectiveness of remedial measures in reducing their annual loadings are important facets of the ‘Upstream Thinking’ project. This, however, relies on the ability to monitor effectively the sporadic releases and fluxes of these chemicals within the catchment.

Currently, most water quality monitoring programmes rely on the collection of low-volume (0.5–1 L) spot (bottle or grab) water samples, usually at monthly, or at most weekly, time intervals. This approach has a number of drawbacks; it is both expensive and time consuming and has the potential to miss sporadic changes in the concentration of contaminants. Use of in situ techniques, such as passive sampling devices, can overcome many of these problems and can be beneficial in investigations where the concentration of a pollutant is known to fluctuate widely (Vrana et al. 2005; Booij et al. 2007). In addition, passive samplers have the advantages of being relatively low-cost, non-mechanical, requiring no power and little maintenance and being easy to deploy in a range of remote field locations. Depending on their length of deployment, such devices can be used to derive the time-weighted average (TWA) concentration of a substance in the sampled medium or the equilibrium concentration of a substance in the sampler. In order to measure TWA concentrations, devices can be used only when the uptake of a compound is time integrative, and this is normally considered as the period up to the half-time to equilibrium (*t*_(0.5)_). In addition, samplers need to be calibrated in the laboratory or field, or by using physicochemical-based models, in order to obtain the uptake rate (*R*_*s*_) of a specific analyte. *R*_*s*_ is normally expressed as the equivalent volume of water cleared per unit time (L day^−1^) for each analyte (Vrana et al. 2005; Booij et al. 2007).

A number of different designs of passive sampler is available, including semi-permeable membranes devices, polymer sheets or Chemcatcher^®^ for non-polar pollutants (Charriau et al. 2016; Lissalde et al. 2016) and the polar organic chemical integrative sampler (POCIS) (Alvarez et al. 2004; Van Metre et al. 2017) o-DGT (Guibal et al. 2017; Challis et al. 2016) and the polar version of the Chemcatcher^®^ (Charriau et al. 2016; Lissalde et al. 2016; Petrie et al. 2016) for polar pollutants. For most non-polar samplers, the off-loading rates of performance reference compounds (PRCs) can be used to adjust *R*_*S*_ for the effects of water temperature and hydrodynamic conditions in the field (Huckins et al. 2002; Booij et al. 2016). The use of PRCs with adsorptive and ion-exchange samplers has been attempted, but only with limited success (Carpinteiro et al. 2016; Fauvelle et al. 2012; Fauvelle et al. 2014; Fauvelle et al. 2017; Harman et al. 2012; Mazzella et al. 2010).There is limited reported use of this technology for the measurement of acidic herbicides and particularly its use at the river catchment scale (Fauvelle et al. 2012; Fauvelle et al. 2014; Kaserzon et al. 2014; Mazzella et al. 2010; Seen et al. 2014; Van Metre et al. 2017). Most workers used POCIS with typically either hydrophilic lipophilic balance (e.g. Oasis^®^ HLB) or strong anion-exchange (e.g. Oasis^®^ MAX) sorbent powders as the receiving phase to measure these analytes. However, there has been concern regarding the loose receiving phase material contained in the POCIS moving and sagging towards the base of the device during field deployments. This gives an unpredictable active sampling surface area and hence uptake rate, and can be associated with poor reproducibly between devices (Mills et al. 2014). This problem can be overcome by the use of an immobilised receiving phase. Tran et al. (2007), Kaserzon et al. (2014) and Novic et al. (2017) used 3M Empore™ solid-phase extraction disks (47-mm diameter SDB-RPS and SDB-XC phases) and Guibal et al. (2017) used Oasis^®^ HLB and Oasis^®^ MAX sorbents embedded in a gel matrix as in the o-DGT to measure a limited number of pesticides with acidic characteristics.

We developed a new variant of the Chemcatcher^®^ that used a 3M Empore™ anion-exchange disk as receiving phase, overlaid with a polyethersulphone (PES) diffusion membrane. The *R*_*s*_ of eight acidic herbicides was measured in the laboratory with a semi-static calibration system using river water as the test medium. The utility of the device was evaluated in two different field investigations in the River Exe catchment, which has a long history of pollution by acidic herbicides. The value of the use of passive samplers in meeting the aims of the ‘Upstream Thinking’ project and in the management of river catchments is discussed.

## Experimental

### Standards, chemicals and reagents

The acidic herbicides and the preparation of stock solutions and related standards and reagents is described in the Electronic supplementary material.

### Analysis of acidic herbicides in water samples

The concentration of acidic herbicides in laboratory and field water spot samples was measured using a validated and accredited UK water industry procedure (Environment Agency 1997). Briefly, water samples (100 mL for laboratory calibration tests and 1 L for field trials) were filtered (47-mm diameter 0.2-μm pore size borosilicate glass micro-fibre filter (Fisher Scientific Ltd., Loughborough, UK)) to remove particulate matter and acidified using hydrochloric acid (pH 1.5–2.0). The samples were extracted using IST ISOLUTE^®^ ENV+ solid-phase cartridges (3 mL) and eluted with ethyl acetate followed by acetone. The acidic herbicides were derivatised using diazomethane and after solvent exchange into iso-octane were analysed by gas chromatography-mass spectrometry (GC-MS) operated in the selected ion-monitoring mode (Table S2). A full description of the analytical method is given in the Electronic supplementary material. Limits of detection for the acidic herbicides are given in Table S2. Recoveries of the acidic herbicides from spiked River Exe water (*n* = 14) ranged from 83 to 99% (standard deviation ± 7–12%). The method was assessed using the UK water industry external Aquacheck (group 8 analytes) quality assurance scheme.

### Preparation and processing of Chemcatcher® samplers

Empore™ Chemcatcher^®^ PTFE bodies (University of Portsmouth, Portsmouth, UK) were cleaned in Decon 90 detergent, water, rinsed with acetone and dried. Receiving phase 3M Empore™ anion-SR exchange disks (47 mm) were from Sigma-Aldrich Co. Ltd. (Gillingham, UK). Before use, disks were pre-conditioned (using a vacuum manifold) with successive aliquots (50 mL) of acetone, methanol, water, 1 M sodium hydroxide and finally water. Conditioned disks were not allowed to dry out prior to use. Disks (47 mm) of polyethersulphone (PES) diffusion membrane (Supor^®^ 200, 0.2-μm pore size) were from Pall Europe Ltd. (Portsmouth, UK). The PES membranes were soaked in methanol overnight to remove manufacturing impurities and rinsed thoroughly with water before use (Guibal et al. 2015). For laboratory calibration tests, devices were prepared with either a 3M Empore™ disk receiving phase or with the receiving phase overlaid with a PES membrane. For the two field tests, only the latter type of device was used. Once assembled, a small quantity of water was added to the well of the device, to ensure the disk remained wet and the PTFE transport lid fitted.

After use, samplers were disassembled and both receiving phase disks and PES membranes analysed (calibration tests only). Both components were rinsed with water and then dried thoroughly (~ 1 h) on a vacuum manifold. Acidic herbicides were eluted (10 mL ethyl acetate/acetic acid solution, 9:1 *v*/*v*), the extract dried with anhydrous sodium sulphate (100 mg) and briefly centrifuged. An aliquot (8 mL) was removed and evaporated to dryness under nitrogen until all traces of acetic acid were removed, to ensure the efficient derivatisation of the acid herbicides. The extract was methylated using a solution of diazomethane in diethyl ether and analysed by GC-MS as described for water sample extracts (see Electronic supplementary material), except that unextracted calibration standards were used for quantification of the analytes.

### Measurement of Chemcatcher® uptake rates for acidic herbicides

**Fig. 1:**
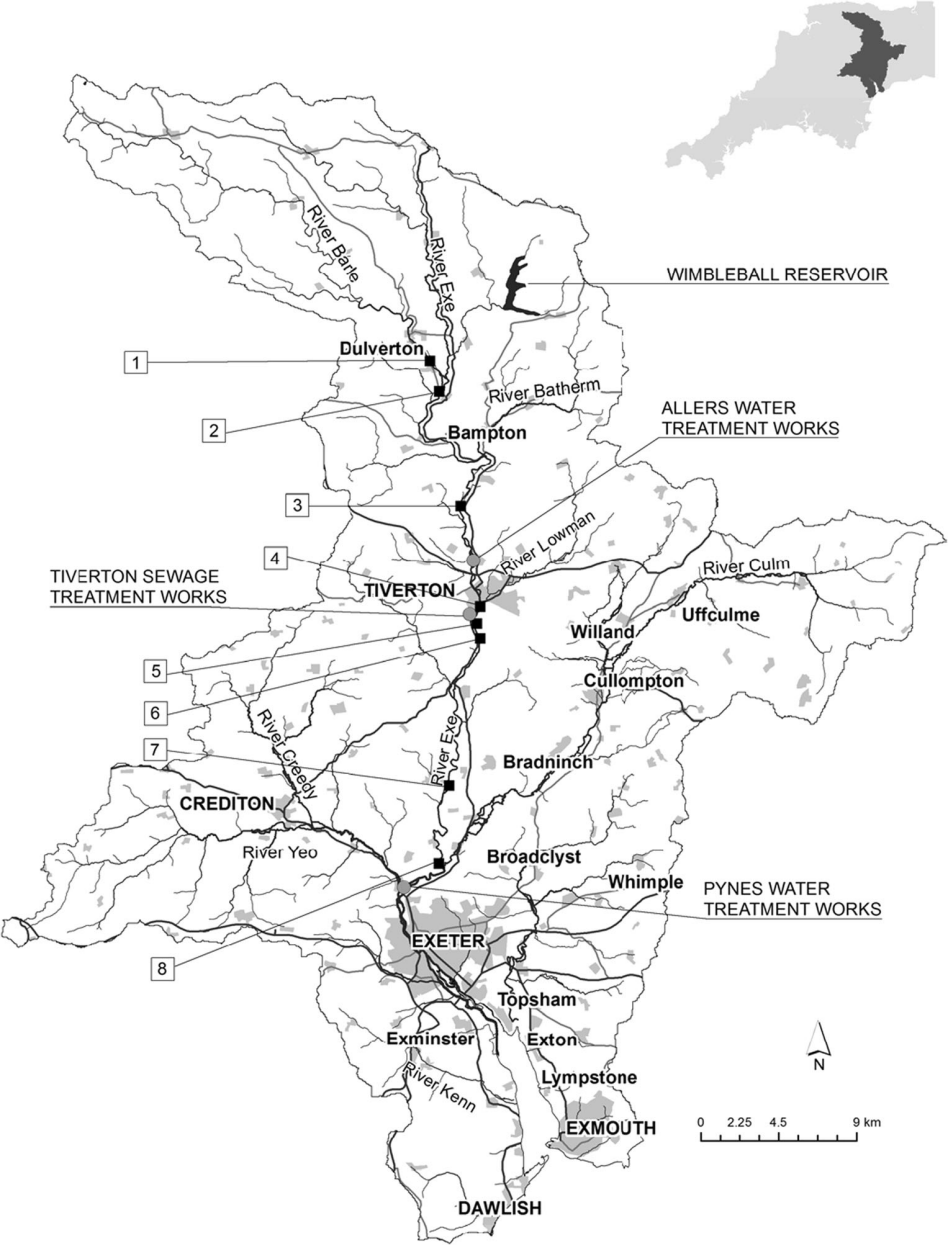
Map of River Exe catchment showing the eight locations for the Chemcatcher^®^ deployments for field trial 1 and the Pynes water treatment works (national grid reference coordinates SX93009710) where raw water was collected for the laboratory uptake rate experiments. The sites are numbered in sequence running down the catchment for ease of visualisation. Key to location of sites, together with national grid reference coordinates: (1) River Barle at Pixton Hill above Brushford sewage treatment works (SS92482625), (2) River Exe at Exebridge pumping station (SS93012447), (3) River Exe at Ironbridge near Stoodleigh (SS94261782), (4) River Lowman at confluence with River Exe (SS95381200), (5) River Exe upstream of Tiverton sewage treatment works (SS95191104), (6) River Exe downstream of Tiverton sewage treatment works (SS95381018), (7) River Exe at Thorverton gauging station (SS93580161), (8) River Exe at Northbridge intake (SX93009710)

In order to simulate monitoring conditions in the field, untreated River Exe water, sampled at the inlet to Pynes Water Treatment Works (national grid reference coordinates SX93009710, Fig. 1), was used as the matrix for the laboratory calibration tests. Typically, water from this area exhibits a relatively high concentration of humic acids, with a dissolved organic carbon content ranging 2–10 mg L^−1^ and a pH ~ 7.5–8.5. A batch (~ 1000 L) of river water was collected and stored in a pre-cleaned vessel on-site at the laboratory. Analysis of the river water showed no detectable concentrations of acidic herbicides (method limits of detection (LoD) in the range 7–11 ng L^−1^). Key water quality parameters are shown in Table S3. Sub-aliquots (~ 20 L) of river water were removed at set periods, allowed to fully equilibrate to room temperature (~ 16–18 °C) and then spiked with 2,4-D, dicamba, dichlorprop, fluroxypyr, MCPA, MCPB, mecoprop and tricolpyr (1 μg L^−1^ each). This concentration was considered to be broadly representative of the maximum level likely to be encountered in the River Exe during field deployments. These eight herbicides were selected as the 3M Empore™ anion-exchange disks retained them efficiently and gave good recoveries using the ethyl acetate/acetic acid eluent (Table S4). In addition, they have a range of physico-chemical properties (Table S5) and have been found to be present historically in the River Exe catchment.

The *R*_*S*_ of the acidic herbicides were measured using a calibration apparatus similar to that described by Vrana et al. (2006) (Fig. S2). In this case, a semi-static system was used rather than a flow through design. Two glass tanks (300 mm × 300 mm × 300 mm), each containing a rotatable PTFE carousel able to hold 14 Chemcatcher^®^ samplers on two layers, were used. The complete rig was pre-conditioned using unspiked River Exe water for 48 h, with the water exchanged daily. One tank held devices fitted with only a 3M Empore™ receiving phase disk, the other with devices also overlaid with a PES membrane. Each tank was filled with spiked (1 μg L^−1^) River Exe water (16 L). The pH of the water was 7.8. Both carousels were stirred (~ 50 rpm) using an overhead stirrer. This gave a linear water velocity over the surface of the samplers of ~ 0.5 m s^−1^, typically representative of flows in the Middle Exe catchment (http://nrfa.ceh.ac.uk/data/station/meanflow/45001). The calibration trial was performed over 16 days with water in each tank being completely drained and rapidly replenished with pre-equilibrated spiked River Exe water every 24 h. The small cavity on top of the samplers ensured that the PES membrane remained completely wet during these transfers. The concentration of the acidic herbicides was measured in both solutions (drained and freshly spiked river water) at each operation. A sampler was removed randomly from each tank after exposure times of 8, 24, 32, 48, 72, 96, 120, 144, 169, 193, 241, 288, 337 and 386 h. A higher frequency of removal was used at the start of the exposure, in order to investigate if there was a lag-phase in uptake. This was important to determine as the PES membrane can accumulate substances, resulting in a delay in their subsequent transfer to the receiving phase disk. A ‘dummy’ Empore™ Chemcatcher^®^ PTFE body was inserted into the resultant vacant position in each carousel in order to maintain consistent hydrodynamics in the tank throughout the study. The temperature of the water in each tank was recorded. A blank Chemcatcher^®^ of each configuration was exposed to the atmosphere whenever manipulations were performed in order to account for background contamination. The mass of each acidic herbicide accumulated in the 3M Empore™ disk and PES membrane at each exposure time was measured using the analytical procedure described above. The mass of each compound versus exposure time was plotted and the uptake curve fitted using the standard exponential function in the non-linear regression routine of GenStat 15 (VSN International Ltd.). The slope of these plots was used to calculate the *R*_*S*_ for each compound. PRCs were not used in the calibration study as such analytes would not be expected to off-load, isotropically, from the anion-exchange sorbent.

### Chemcatcher® field deployments in River Exe catchment

Two field trials were carried out in the River Exe catchment during late spring/early summer 2013. Previous data indicated sporadic inputs of acidic herbicides into the catchment during these months. The first field trial was conducted to assess the performance of the Chemcatcher^®^ alongside repeated high-frequency spot water sampling; the second to investigate if the passive sampler could provide information on which specific tributaries were primarily responsible for pollution by acidic herbicides in the lower Exe catchment. Such data would help to target future ‘on-farm’ initiatives within the ‘Upstream Thinking’ project with the aim of reducing overall loadings of acidic herbicides in the catchment.

- *Field trial 1* was conducted during 8–22 May 2013 at eight locations that encompassed a range of land uses and riverine conditions down the River Exe catchment (Fig. 1). Triplicate Empore™ Chemcatcher^®^ devices were mounted in a bespoke stainless steel cage (Fig. S3) and securely attached to a mooring on the river bank using a chain. The deployment period for all samplers fell within the range 334.5 ± 1.5 h (the precise exposure time for each device being recorded accurately). A field blank Chemcatcher^®^ was exposed at each site during deployment and retrieval and handled subsequently as the experimental samplers. After retrieval, the cavity in the body of the samplers was filled with River Exe water, devices sealed, transported to laboratory in cool boxes and stored in the dark at 2–8 °C until analysis. The anion-exchange receiving phase disks were removed and processed as above in order to determine the masses of each acidic herbicide accumulated. Acidic herbicides were not measured in the PES membranes, as the previous laboratory calibration experiments we undertook in this study showed that this polymeric material did not retain them.

During the deployment, a spot sample of river water (1 L) was collected (borosilicate glass bottle with screw cap containing a PTFE insert) at each location on seven occasions (corresponding to days 0, 2, 5, 7, 9, 12 and 14 of the study), with precise sampling times being recorded. Water samples were returned to the laboratory, filtered (0.2-μm filter), acidified (pH 1.5–2.0) with HCl and refrigerated (4 °C) until analysis, not exceeding 14 days. Acidic herbicides were analysed as described above. In addition, at these times of collection, water temperature, pH, nitrate, dissolved organic carbon (DOC), and where feasible, the river water flow velocity were measured.

- *Field trial 2* was conducted during 18 June–4 July 2013 at nine locations within the Exe catchment, including an additional six sites not examined in the first field trial (Fig. S4). Duplicate Empore™ Chemcatcher^®^ samplers were deployed as above for an average period of 382 ± 2 h, with their precise time of exposure recorded accurately at each site. A single Chemcatcher^®^ field blank was exposed at each site during sampler deployment and retrieval. A spot water sample was taken at each site only during deployment (day 0) and retrieval (day 16) of the devices, together with pH and temperature. Laboratory analyses of the acidic herbicides in the anion-exchange disks and spot water samples were undertaken as above.

### Theory of passive sampling

The theory of the uptake of a chemical by a passive sampler has been described previously (Vrana et al. 2005; Booij et al. 2007; Huckins et al. 2006). Consequently, only the key equation used in this study is given. The uptake of a chemical by a device over the period between the start of exposure and the half-time to equilibrium (*t*_(0.5)_) is approximately linear (integrative mode) and can be described by the following:

1 $$ {M}_S(t)={C}_w{R}_St $$

where *M*_*s*_(*t*) is the mass (ng) of analyte in the sampler after exposure time *t* (day), *C*_*w*_ is the concentration (ng L^−1^) of analye in the water and *R*_*s*_ is the sampler uptake rate (L day^−1^). In laboratory calibration studies, *R*_*s*_ can be calculated from Eq. 1 using the slope (*M*_*s*_(*t*)/*t*) of the regression of the mass in the sampler on time (over the linear portion of the uptake data) and the concentration (*C*_*w*_) in the water. Once *R*_*s*_ is known, this can be used in field trials to calculate *C*_*w*_ which corresponds to the TWA concentration of the chemical over the deployment period (*t*).

## Results and discussion

### Selection and analysis of acidic herbicides

Initially, the recovery of 12 acidic herbicides was evaluated (Table S2). The disk retained all the compounds, but with variable recoveries (17–93%) (Table S4). The evaporation step was expected to cause losses of the more volatile compounds. The strong acids (*pK*_*a*_ = 1.9–2.3) (Table S5) gave poor recoveries, probably due to formation of strong ion-pairs. As the eluent contained acetic acid (p*K*_*a*_ 4.76), these strong ion pairs were not sufficiently disrupted to allow the strongly acidic herbicides to be quantitatively eluted. A more limited set of eight acidic herbicides was taken forward for the laboratory uptake experiments.

### Laboratory uptake experiments

Due to the high polarity and aqueous solubility of the acidic herbicides (Table S5), they were expected to be freely dissolved. Natural water was used to replicate field conditions particularly the concentration of dissolved organic carbon and nitrate, which can be high in this catchment. At the pH of the calibration water (pH = 7.8), all analytes were totally in their ionic form (Table S5). The concentration of each acidic herbicide measured in the river water was below the detection limit of the analytical method.

Using the semi-static calibration system, the concentrations of all the acid herbicides in the two tanks remained reasonably constant. The concentrations in the tanks varied by between ± 10% (for dicamba) and ± 24% for fluroxypyr, and fell around ± 13% for most of the other test compounds over the 16-day deployment. By replacing the spiked calibration water every ~ 24 h, the Chemcatcher^®^ samplers were effectively exposed to a nominal concentration of ~ 1 μg L^−1^ of each acidic herbicide over the whole laboratory trial. Paired sample *t* tests showed that there were no significant differences between the concentrations of the acidic herbicides, with the exception of triclopyr, in the water in calibration tanks for the disks with and without membranes. Further details are given in Table S6(a). Uptake of test analytes by the samplers was at a maximum on day 1 (14 devices in carrousel). Using the highest *R*_*s*_ value (mecoprop, Table 1), this resulted in 1.58 L of water being taken up by the samplers. This value corresponded to ~ 10% of the total volume (16 L) of water in the tank, being in the range acceptable for passive sampler calibration studies using static or semi-static systems (Stephens and Müller 2007). This effect during the first few days of the laboratory trial probably accounted for the higher variabilities found in the concentrations of the analytes in the tank. The water temperature (15.8–18.5 °C) and velocity (0.5 m s^−1^) remained relatively constant in both tanks during the experiment.

**Table 1:** Half time to equilibrium (*t*_0.5_) and uptake rates (*R*_*s*_) and associated uncertainty (± Δ) for the eight acidic herbicides using a Chemcatcher^®^ fitted 47 mm 3M Empore™ anion-exchange disk overlaid with a 47-mm PES membrane. A laboratory semi-static calibration system was used, nominal aqueous concentration of analytes was ~ 1 μg L^−1^, water temperature ranged between 15.8 and 18.5 °C and water velocity was ~ 0.5 m s^−1^. The estimated *R*_*s*_ for a standard 47 mm diameter POCIS are given for comparison. Also see Tables S6(a-c) for further information

Acidic herbicide	*t*_(0.5)_ (days)	*R*_*s*_ (± Δ) (L day^−1^)	Estimated *R*_*s*_ for POCIS (L day^−1^)
2,4-D	17.0	0.064 (0.009)	0.193
Dicamba	16.0	0.044 (0.007)	0.132
Dichlorprop	6.3	0.112 (0.024)	0.337
Fluroxypyr	6.4	0.073 (0.018)	0.220
MCPA	58.0	0.062 (0.009)	0.187
MCPB	11.0	0.062 (0.013)	0.187
Mecoprop	6.0	0.113 (0.025)	0.340
Triclopyr	8.5	0.053 (0.010)	0.160

**Fig. 2:**
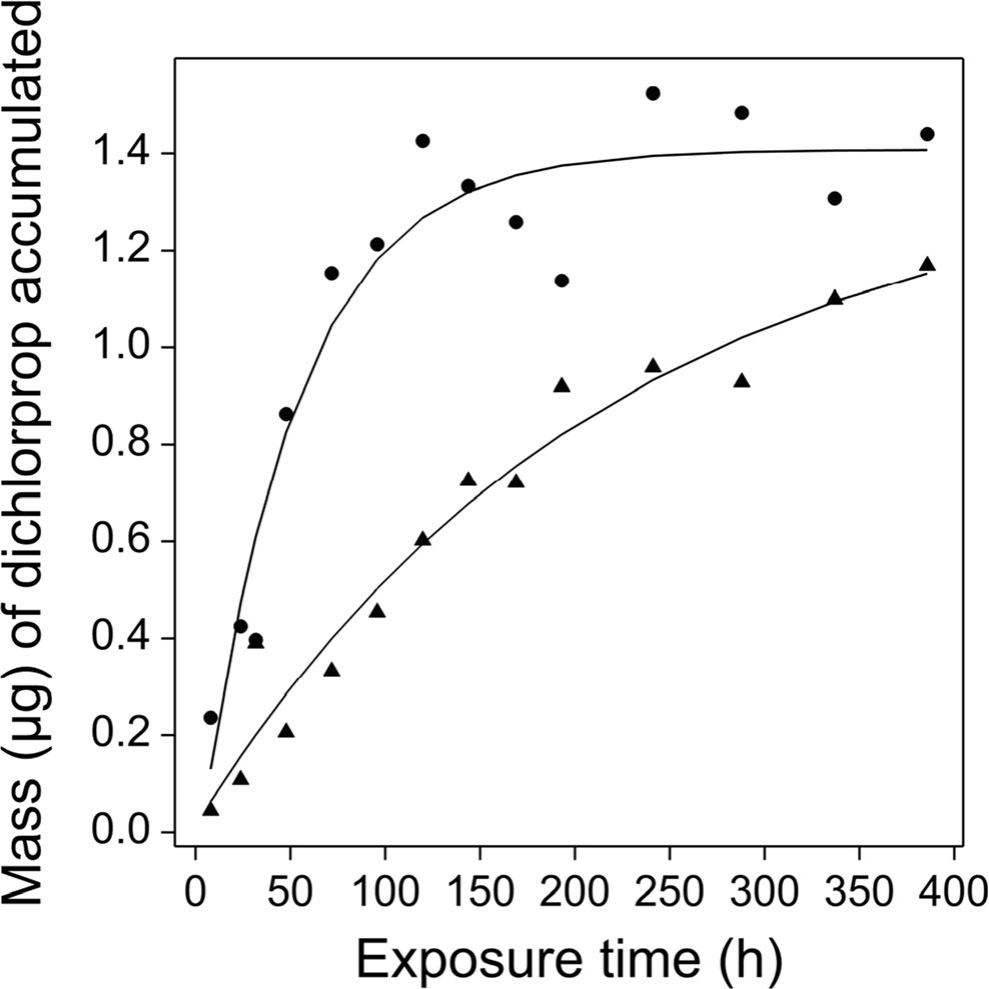
Uptake curves for dichloroprop measured in the laboratory calibration tests using Chemcatcher^®^ devices fitted with an overlaying PES membrane (triangles) and no PES membrane (circles). Curves were fitted using the standard exponential function in the non-linear regression routine of GenStat 15 (VSN International Ltd.)

All the acidic herbicides could be measured satisfactorily in extracts obtained from the 3M Empore™ anion-exchange disks from Chemcatcher^®^ devices with and without an overlying diffusion membrane using the GC-MS procedure. The amount of acidic herbicide measured in the laboratory blank samplers that were exposed during the deployment and retrieval operations was below the detection limit of the analytical method. Figure 2 shows the uptake curves for dichlorprop by the sampler with and without a PES membrane. Uptake curves for the seven other analytes (both versions of the device) are shown in Fig. S5. For all the compounds, there was no identifiable lag-phase in uptake by the devices fitted with the diffusion membrane. This was supported by analysis of the PES membranes, as no measureable amounts of any herbicide, other than traces of fluroxypyr (~ 6 ng), were found over the time course of the laboratory uptake experiment. Estimated LoD was 2 ng/analyte per disk or PES membrane. The absence of a lag-phase for ionisable compounds was in agreement with the findings of Fauvelle et al. (2012) when using POCIS fitted with PES membranes. However, recently, Endo and Matsuura (2018) have shown that an ionic compound (2-nathphalene sulfonate) could be retained by a PES membrane and further work in this area is needed.

There was more variability in the uptake data for the samplers without a PES membrane. In the absence of a membrane, higher *R*_*s*_ values (data not shown) were obtained and equilibrium was approached more rapidly, thus limiting the period during which TWA concentrations of analytes can be measured in field deployments. Although passive samplers based on the Chemcatcher^®^ principle can be used without a PES membrane (Fernandez et al. 2014; Ahkola et al. 2017), in our study, after about 7-day exposure, the structural integrity of the 3M Empore™ anion-exchange disks was starting to be compromised making subsequent handling and processing difficult. The naked disks also became highly stained with fulvic and humic substances present in the river water. Hence, subsequent laboratory investigations and field deployments were limited to devices fitted with an overlying protective PES membrane. However, samplers fitted with only a disk maybe useful in short-term (e.g. 1–4 days) investigative or forensic field studies looking for the presence or absence of an acidic herbicide.

The uptake parameters for the eight acidic herbicides are shown in Table S6(b). The time integrative period *t*_(0.5)_ for the acid herbicides varied between 6.0 to 58.0 days. Apart from MCPA, all the analytes had roughly similar (*t*_0.5_) values (Table 1). The reason for the higher *t*_0.5_ for MCPA is not known. The *R*_*s*_ (L day^−1^) values were calculated based on the time interval (*t*_0.5_) to half equilibrium and using the mean aqueous concentration of test analyte over this period (Table S6(c)). At the water temperature range and turbulence tested, the *R*_*s*_ values ranged from 0.044 to 0.113 L day^−1^ (Table 1). Based on linear regression analyses using varying numbers of time points above the *t*_(0.5)_, it would be possible to extend the range of times used in estimating the slope of the linear region of the calibration curve for most compounds without a marked effect on the value of the slope: dichlorprop (from 6 to 11 days), fluroxypyr (from 6 to 10 days), mecoprop (from 6 to 11 days), triclopyr (from 8 to 11 days). For dicamba, it would not be possible to extend the exposure time used. Longer deployments of the Chemcatcher^®^ within a catchment could be used forensically to detect the presence or absence of a given herbicide. Unlike many persistent non-polar organic pollutants, acidic herbicides are present in surface waters often sporadically, and this is related to their seasonal application and rainfall events. Longer field deployments (e.g. ≥ 14 days) may therefore not be warranted for this class of pollutants.

A relationship between *R*_*s*_ and physicochemical (e.g. log *K*_*ow*_ or *pK*_*a*_) properties of this limited set of acidic herbicides was not apparent (data not shown). Other workers have also found no direct simple relationship for uptake of polar compounds by passive samplers based on a single physicochemical property (Miller et al. 2016; Morin et al. 2018).

The only comparable data on the uptake of acidic herbicides (2,4-D, dicamba, dichlorprop, MCPA and mecoprop) by a passive sampler (POCIS) using an anion-exchange receiving phase (Oasis^®^ MAX) is that of Fauvelle et al. (2012). Using drinking water and river water (spiked with 50 mg L^−1^ of nitrate) as the test matrices, *t*_0.5_ ranged from 17 to > 21 days. The higher *t*_0.5_ values found are probably related to the larger mass of sorbent material used in the POCIS. *R*_*s*_ for the herbicides in their study ranged from 0.089 to 0.149 L day^−1^ (Fauvelle et al. 2012). The ratio of the active surface sampling area of the Chemcatcher^®^ (15.2 cm^2^) to the POCIS (45.8 cm^2^) is ~ 3.01. This gave equivalent *R*_*s*_ values for the Chemcatcher^®^ ranging from 0.132 to 0.340 L day^−1^ (Table 1). The relatively higher sequestration rates for the Chemcatcher^®^ may be related to differences in sampler geometries, use of either loose or bound receiving phase materials and the functionality of the anion-exchange materials used.

When using an anion-exchanger as a receiving phase any negatively charged compounds present in the water column may also be sequestered. This may be problematic for nutrients such as nitrates (NO_3_^−^) which can be present at high concentrations and thereby reduce the number of available ion-exchange sites by competition and hence the uptake capacity of the sampler. This effect was investigated by Fauvelle et al. (2012) using river water spiked with a high concentration (50 mg L^−1^) of nitrate. Using the Oasis^®^ Max sorbent, there was no influence of nitrate on the uptake of analytes. In our experiments, the presence of nitrate (13.5 mg L^−1^) in the River Exe water was, therefore, unlikely to affect the uptake of the acidic herbicides.

### Field trial 1

This 14-day field trial investigated the performance of the Chemcatcher^®^ sampler alongside frequent (2–3 days collections) spot water sampling at eight locations in the River Exe catchment (Fig. 1). The sites selected covered a broad transect of the River Exe together with some tributaries (Barle and Lowman). The aim was to ascertain if the two monitoring approaches gave comparable data on the concentrations of the acidic herbicides and if this combined information could have a role in developing improved river catchment management plans.

Water quality properties and flow characteristics were measured at seven occasions over the deployment period (Table S7(a-h)). As expected, during the field trial, environmental conditions in the river water down the catchment varied (temperature, 8.5–15.4 °C; pH, 6.75–8.67; DOC, 1.0–7.5 mg L^−1^; nitrate, 1.6–34.0 mg L^−1^; flow, 0.20–2.60 m s^−1^). Rainfall during the field trial period varied from 0.0 to 22.5 mm, peaking on 14 May 2013, followed by a prolonged dry period (Fig. S6). These meteorological conditions were responsible, in part, for the variability in the water properties found during the field trial. The peak concentration of nitrate found during the field trial was unlikely to affect the effective sequestration of the acidic herbicides (Fauvelle et al., 2012).

The concentration of acidic herbicides found in spot water samples during the field trial are given in Table S7(a-h), with an example at site 4 (River Lowman at confluence with River Exe) shown in Table 2. At locations further up the catchment (sites 1–3) the concentration of all herbicides was generally below the analytical limit of detection. Elevated concentrations of MCPA and mecoprop were found at site 4 and this persisted to a lesser extent at sites 5–8. On two occasions the concentration of mecoprop (day 0 = 868 ng L^−1^ and day 14 = 144 ng L^−1^) exceeded the PCV (EU Council Directive 1998). After the rainfall event on day 7 (14 May 2013), elevated concentrations of 2,4-D, dicamba, MCPA and mecoprop were often found in the catchment. Understanding the dynamics of the catchment in response to stochastic events is complex, and it is difficult to link directly concentrations of acidic herbicides found in the river to rainfall as there is a number of additional influential factors (e.g. method and application rates of herbicides, croppage, field slope and drainage, soil type and moisture deficit) to be considered.

**Table 2:** Aqueous concentration and estimated time-weighted average (TWA) concentration measured by the Chemcatcher^®^ (CC) (*n* = 3) for eight acidic herbicides in field trial 1 at site 4 (River Lowman at confluence with River Exe (national grid reference coordinates SS95381200)). The concentration of acidic herbicides found in the Chemcatcher^®^ field blanks was below the detection limit of the analytical method. Data for the other sites in field trial 1 are given in Tables S7(a-h)

Acidic herbicide	Concentration (ng L^−1^) in spot water samples							CC 1 TWA^a^ (ng L^−1^)	CC 2 TWA^a^ (ng L^−1^)	CC 3 TWA^a^ (ng L^−1^)	Average TWA^a^ (ng L^−1^)
	Day 0	Day 2	Day 5	Day 7	Day 9	Day 12	Day 14				
2,4-D	< 7	< 7	< 7	< 7	< 7	< 7	< 7	4	4	4	4
Dicamba	< 7	< 7	< 7	< 7	< 7	< 7	< 7	6	7	4	6
Dichlorprop	< 8	< 8	< 8	< 8	< 8	< 8	< 8	< 1	< 1	< 1	< 1
Fluroxypyr	< 11	< 11	< 11	< 11	< 11	< 11	< 11	6	6	5	6
MCPA	< 8	< 8	< 8	*35*	< 8	*19*	< 8	*12*	*12*	*13*	*12*
MCPB	< 9	< 9	< 9	< 9	< 9	< 9	< 9	< 1	< 1	< 1	< 1
Mecoprop	*868*	*25*	< 7	*53*	*36*	*21*	*144*	*68*	*71*	*65*	*68*
Triclopyr	< 8	< 8	< 8	*17*	< 8	*10*	< 8	*14*	*14*	*15*	*14*

^a^Estimated TWA concentration assuming the three Chemcatcher^®^ samplers (CC) were in the time integrative mode for all eight compounds over the 14-day deployment. Amount of acidic herbicide found in the associated field blanks taken into consideration when calculating the TWA concentration. Italicized entries show elevated concentrations

The TWA concentrations for the acidic herbicides were calculated (Eq. 1) assuming that the samplers remained in the integrative mode over 14 days (data given in Table 2 and Table S7(a-h)). No acidic herbicides were detected in the field blanks. It is recognised that dichlorprop, fluroxypyr and mecoprop would be in the curvilinear phase of uptake by the end of the field trial, so this approach is not strictly valid. However, due to the highly sporadic nature of the inputs of acidic herbicides into the catchment, with their presence in the water column associated with rainfall events, a 2-week deployment was thought to give the best opportunity of detecting these substances at measurable concentrations. There were differences in the conditions used for the laboratory uptake rate experiment and those appertaining during the field trial. These differences will affect the *R*_*s*_ values for the acidic herbicides. However, the effect of water temperature and flow on the uptake of a range of polar analytes by the POCIS has been shown to be relatively small (Li et al. 2010). A solution to overcome this problem is the use of PRCs, but as their effectiveness with polar passive samplers is not fully proven, alternative solutions such as passive flow monitors (e.g. rate of dissolution of calcium sulphate casts) has been proposed (Fauvelle et al. 2017). There was limited biofouling of the PES membrane over the deployment, but some staining was observed due to the presence of humic and fulvic substances in the river water. Generally, there was good reproducibility (< 11% variability in the amount of acidic herbicide sequestered) between the three individual Chemcatcher^®^ samplers in the same cage at the same site in terms of the mass of herbicide sequestered. This is an advantage of use of an immobilised receiving phase in the form of disk, as material cannot be displaced during field deployments. This is a recognised drawback of the use of a loose solid-phase extraction matrix as receiving phase in the POCIS (Mills et al. 2014).

It is difficult to link directly concentrations measured by the two monitoring techniques, as there is no information on the variability of concentration of pollutants during the intervals (2–3 days) between the spot samples. This is compounded by the likely sporadic inputs of acidic herbicides along the catchment. However, where higher concentrations were found in water samples this was also reflected in higher TWA concentrations being measured by the Chemcatcher^®^. Table 3 shows a comparison between the detection of the eight acidic herbicides found using the two methods. There was broad agreement between the two approaches; however, passive samplers were able to detect the presence of acidic herbicides on more occasions over the trial period. It was evident that the burden of pollution increased downstream in the catchment.

**Table 3:**
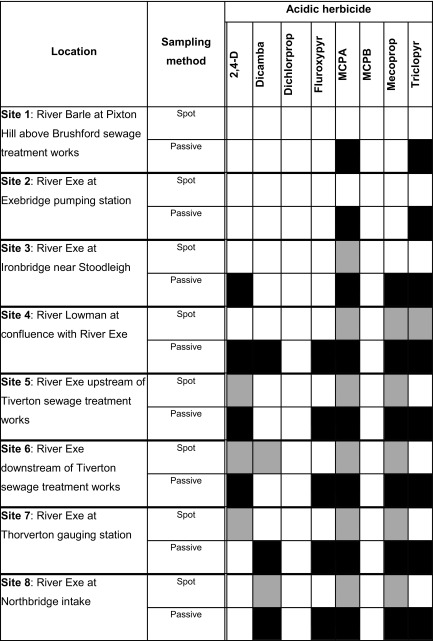
Comparison of spot water sampling (grey coloured box) and passive sampling (Chemcatcher^®^) (black coloured box) techniques for the detection of eight acidic herbicides at the eight field trial 1 sites (see Fig. 1) in the River Exe catchment

### Field trial 2

This trial was undertaken to study if the Chemcatcher^®^ could be used as an investigative tool to detect highly sporadic inputs of acidic herbicides not revealed by routine less frequent (weekly or monthly) spot sampling methods. Such an approach is typically used by the water industry and environment agencies for their regulatory monitoring requirements. Duplicate samplers were deployed for 16 days at nine sites, including six locations within the catchment not investigated during field trial 1 (Fig. S4). Spot samples of water were collected on deployment (day 0) and retrieval (day 16) of the devices with water temperature (14.2–18.2 °C), pH (7.17–8.38) and flow (0.10–0.50 m s^−1^) recorded. These field conditions were broadly within the temperature range and flow velocity used in the laboratory uptake experiment.

The concentration of acidic herbicides found in spot water samples during the field trial are shown in Table S8(a–i), with an example at site 2 (Calverleigh Stream at Lower Farleigh) shown in Table 4. At most sites, there was only limited environmental impact by the nine acidic herbicides and this was in accord with the findings of field trial 1. However, a significant pollution incident was identified at site 2 with highly elevated concentrations of fluroxypyr (2089 ng L^−1^) and triclopyr (5029 ng L^−1^) found. These were ~ 20 and 50 times above the PCV respectively and represented a major pollution incident in the catchment. Calverleigh Stream is a small tributary draining mostly a dairy farming area; it joins the River Exe just below the Allers water treatment works. It was evident that large quantities of pyridine-based acidic herbicides were being used to control pernicious weeds in grassland.

**Table 4:** Aqueous concentration and estimated time-weighted average (TWA) concentration measured by the Chemcatcher^®^ (CC) (*n* = 2) for eight acidic herbicides in field trial 2 at site 2 (Calverleigh Stream at Lower Farleigh (national grid reference coordinates SS93111452)). The concentration of acidic herbicides found in the Chemcatcher^®^ field blanks was below the detection limit of the analytical method. Data for the other sites in field trial 2 are given in Tables S8(a–h)

Acidic herbicide	Aqueous concentration (ng L^−1^) in spot water samples		CC 1 TWA^a^ (ng L^−1^)	CC 2 TWA^a^ (ng L^−1^)	Average TWA^a^ (ng L^−1^)
	Day 0	Day 16			
2,4-D	< 7	< 7	< 1	< 1	< 1
Dicamba	< 7	< 7	< 1	< 1	< 1
Dichlorprop	< 8	< 8	1	1	1
Fluroxypyr	*2089*	*27*	*57*	*66*	*61*
MCPA	< 8	< 8	*153*	*183*	*168*
MCPB	< 9	< 9	< 1	< 1	< 1
Mecoprop	< 7	< 7	2	2	2
Triclopyr	*5029*	*24*	*138*	*158*	*148*

^a^Estimated TWA concentration assuming the Chemcatcher^®^ samplers (CC) were in the time integrative mode for all eight compounds over the 16-day deployment. Italicized entries show elevated concentrations

The estimated TWA concentrations of the acidic herbicides were calculated (Eq. 1) assuming linear uptake over the 16-day deployment. The values are shown in Table S8(a-i), with an example at site 2 shown in Table 4. No acidic herbicides were detected in the field blanks; there was limited biofouling of the PES membrane. As in field trial 1, there was a general agreement between the two methods in terms of the range of analytes detected those that could be quantified. Looking at the pollution incident at site 2, an elevated TWA concentration of MCPA (average value = 168 ng L^−1^) was found using the Chemcatcher^®^ but this was missed using the infrequent spot sampling approach. This 16-day TWA value was above (~ 1.7 times) the PCV, indicating that there must have been an even larger input of MCPA occurring at some point over the field trial. MCPA is one of the most highly used acidic herbicides in the South West Water Ltd. Region (Table S1). The pollution event is expected to be unrelated to the significant inputs of fluroxypyr and triclopyr that were also found in both water samples and by the Chemcatcher^®^. Both these events are of possible concern with respect to the Pynes water treatment works further downstream in the catchment. Here, these residues may get into treated drinking water supplies if the granular activated carbon (GAC) contactor plant was not capable of fully removing these pesticides. It should be noted that this design of Chemcatcher^®^ was also found to be suitable for the detection of other acidic pesticides including benazolin, bentazone, bromoxynil, clopyralid, 2,4-DB, ioxynil, picloram. These are used to varying degrees for similar agricultural purposes as the eight compounds covered by this study and hence are also sometimes detected in river catchments, including the River Exe.

This field trial shows the effectiveness of the passive sampling approach in pinpointing diffuse sources of pollution and in assisting with catchment management initiatives such the ‘UpStream Thinking’ project (SWW 2017). Here, specific farming areas responsible for pollution events can be identified and various remediation strategies and incentives put in place to limit environmental impacts. It is expected in the future that passive samplers will become increasingly used in regulatory water quality programmes, especially for investigative monitoring activities within the remit of the European Union’s Water Framework Directive (Poulier et al. 2014; Jones et al. 2015). Work in this area for detecting and measuring a wide range of key substances is already proving successful and leading to a better knowledge of the transport and fate of pollutants within surface water systems (Lissalde et al. 2014; Miège et al. 2015; Poulier et al. 2015; Van Metre et al. 2017). Key, however, is an improved understanding of the comparability of different sampling strategies (spot, event triggered or passive) particularly for pollutants (e.g. acidic herbicides, metaldehyde) that are present episodically in river catchments (Roll and Halden 2016).

## Detection of acidic pharmaceuticals

Derivatised solvent extracts obtained from Chemcatcher^®^ deployments in field trial 1 were also reanalysed by a routine GC-MS pollutant screening method used in the South West Water Ltd. laboratory. Three acidic pharmaceuticals, diclofenac, ibuprofen and naproxen that are widely used as non-steroidal anti-inflammatory drugs, were also found to be retained by the 3M Empore™ anion-exchange disk. These substances have *pK*_*a*_ values comparable with some of the acidic herbicides investigated (Table S5). A selected ion monitoring GC-MS method (Table S9) was used to quantify the mass of each of these drugs on the disk (*R*_*S*_ values for these compounds were not available). Table S10 shows their detection pattern at four key sites in field trial 1. Higher amounts of acidic pharmaceuticals were found in extracts obtained from samplers deployed (sites 6 and 8) immediately downstream of the outfall from Tiverton sewage treatment works. Non-steroidal anti-inflammatory drugs are frequently found in surface waters that receive discharges from sewage treatment works (Lindqvist et al. 2005). This configuration of the Chemcatcher^®^ maybe prove useful in monitoring this class of pharmaceuticals, as well other anionic chemicals, as these are often difficult to retain on other types of passive sampling devices that contain non-ionic receiving phases.

## Conclusions

A new variant of the Chemcatcher^®^ passive sampler was developed and calibrated in the laboratory for a range of high use and problematic acidic herbicides. Their uptake into the receiving phase was rapid, with negligible amounts being retained by the PES membrane. Under the conditions tested, the device can measure TWA concentrations over periods of 6–58 days, with *R*_*s*_ values ranging from 0.044 to 0.113 L day^−1^. Some of the *t*_0.5_ values can be extended with minimal effect on the reliability of the estimated TWA concentrations. For deployments of a week, the device would effectively sample ~ 0.3–0.8 L of water. This compares typically to 0.5–1.0 L of water collected and analysed when using routine spot sampling procedures. The GC/MS analytical procedure could measure the acidic herbicides at ~ 1 ng/disk. Over a weekly deployment, the Chemcatcher^®^ should be able to detect acidic herbicides at concentrations 1.3–3.2 ng L^−1^.

In this initial ‘proof of concept’ study, only one water temperature and turbulence was used in the calibration tanks; further work is required to investigate how these environmental parameters and others (e.g. biofouling, DOC and other anionic substances present in the water) affect *R*_*s*_. In addition, the sequestered acidic herbicides are easy to extract from the 3M Empore™ anion-exchange receiving phase disk and the resultant extract is compatible with routine analytical methods (GC/MS or LC/MS) currently in use within the water industry. The efficient extraction of very strongly acidic compounds (e.g. clopyralid) may, however, require further work. This design of sampler may also be useful to measure acidic pharmaceuticals and other pesticides with acidic physicochemical properties in water. In the future, by evaluating the Horizon Atlantic™ version of the Chemcatcher^®^ (Petrie et al. 2016), it may be possible to increase the mass (e.g. 200–500 mg) of sorbent used in the receiving phase and thereby increase the *t*_0.5_ values for the acidic herbicides (Fauvelle et al. 2014). Furthermore, this also may allow the possibility of using mixed immobilised sorbents as receiving phases as has been proposed for the POCIS (e.g. different bi- and tri-phasic sorbent mixtures) (Fauvelle et al. 2012; Iparraguirre et al. 2017) and thereby simultaneously sequester a wider range of polar analytes. Work on this design of Chemcatcher^®^ is currently ongoing using an immobilised layer of Oasis^®^ HLB and Oasis^®^ MAX as a biphasic receiving phase for sequestering polar pollutants.

Two targeted field trials showed the potential value of using passive sampling devices alongside spot water sampling to improve monitoring and hence facilitate a better understanding of river systems. We showed that the Chemcatcher^®^ was able to detect the same range of acidic herbicides found in spot water samples taken regularly over the deployment period. The value of the approach was clearly shown by detection of a high concentration of MCPA missed by spot sampling, probably due to an episodic input of herbicide into the catchment. However, both approaches give complementary data for the management of river catchments and for the effective targeting of remediation programmes.

Chemcatcher^®^ is now beginning to be used in other rivers in the UK to monitor acidic herbicides and to assist in the development of catchment scale management plans. Data from these field trials will be reported at a later date.

## Electronic supplementary material

ESM 1 (DOCX 5128 kb)
