# Supplementary material for: Calibration and application of the Chemcatcher® passive sampler for monitoring acidic herbicides in the River Exe, UK catchment
Source: Environ Sci Pollut Res Int. 2018 Jun 25;25(25):25130–42. doi: 10.1007/s11356-018-2556-3 (PMC6133114; doi:10.1007/s11356-018-2556-3)
Supplement: Supplementary file 1 — (DOCX 5128 kb) [file 11356_2018_2556_MOESM1_ESM.docx]

**Electronic supplementary material**

**Calibration and application of the Chemcatcher^®^ passive sampler for monitoring acidic herbicides in the River Exe, UK catchment**

**Ian Townsend,^a^ Lewis Jones,^a^ Martin Broom,^a^ Anthony Gravell,^b^ Melanie Schumacher,^b^ Gary R. Fones,^c*^ Richard Greenwood^d^ and Graham A. Mills^e^**

^a^South West Water Ltd., Peninsula House, Rydon Lane, Exeter, Devon, EX2 7HR, UK

^b^Natural Resources Wales, NRW Analytical Services at Swansea University, Faraday Building, Swansea University, Singleton Campus, Swansea, SA2 8PP, UK

^c*^School of Earth and Environmental Sciences, University of Portsmouth, Burnaby Road, Portsmouth, PO1 3QL, UK

^d^School of Biological Sciences, University of Portsmouth, King Henry I Street, Portsmouth, Hampshire, PO1 2DY, UK

^e^School of Pharmacy and Biomedical Sciences, University of Portsmouth, White Swan Road, Portsmouth, Hampshire, PO1 2DT, UK.

*Corresponding author: E-mail: [gary.fones@port.ac.uk](mailto:gary.fones@port.ac.uk); Tel: +44 2392 842252; Fax: +44 2392 842244

**Introduction**

The pie chart (Fig. S1†) shows the land use within the South West Water Ltd. Region for the abstraction and supply of drinking water. Latest available data for woodland were taken from (<http://www.woodland-directory-sw.org.uk/index.php?page=south-west-forestry-statistics>) and all other uses from (<https://www.gov.uk/government/statistical-data-sets/structure-of-the-agricultural-industry-in-england-and-the-uk-at-june>).


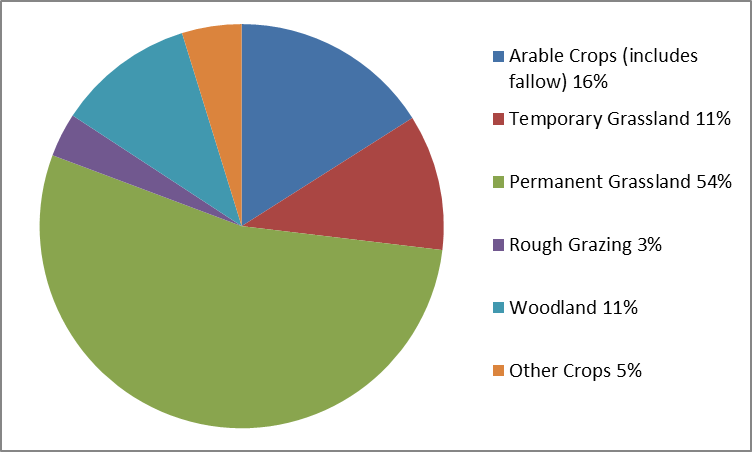


Fig. S1 Land usage in the South West Water Ltd. region (2010)

Table S1 shows the usage of pesticides in the South West Water Ltd. Region. The data for 2011 were obtained commercially by the company using the i-Map Pesticide Database supplied by GFK Kynetec Ltd. (Newbury, Berkshire, UK).

Table S1 Twenty most heavily applied pesticides in the South West Water Ltd. region in 2011 (acidic herbicides are identified in bold type face)

| **Ranking** | **Pesticide** | **Tonnes applied** |
| --- | --- | --- |
| 1 | Glyphosate | 66.7 |
| **2** | **MCPA** | **59.3** |
| 3 | Chlormequat | 47.7 |
| **4** | **Mecoprop/Mecoprop-P** | **29.2** |
| 5 | Chlorothalonil | 27.5 |
| 6 | Pendimethalin | 26.9 |
| 7 | Prosulfocarb | 23.4 |
| **8** | **Triclopyr** | **17.2** |
| **9** | **MCPB** | **14.9** |
| 10 | Chlorotoluron | 12.6 |
| 11 | Prothioconazole | 10.1 |
| 12 | Asulam | 9.4 |
| **13** | **2,4-D** | **8.3** |
| **14** | **Fluroxypyr** | **7.1** |
| 15 | Propamocarb Hydrochloride | 7.1 |
| **16** | **Clopyralid** | **5.7** |
| 17 | Mancozeb | 5.5 |
| 18 | Spiroxamine | 5.0 |
| 19 | Epoxyconazole | 4.4 |
| 20 | Flufenacet | 4.4 |

**Analytical method used for the extraction and analysis of acidic herbicides in water samples**

Water samples (100 mL for laboratory calibration tests and 1 L for field trials) were collected in pre-cleaned borosilicate glass bottles with screw caps and PTFE liners. Particulate matter was removed by filtration under vacuum through a 47 mm glass fibre membrane (1.6 µm pore size, Fisher Scientific UK Ltd., Loughborough, UK). Samples were then acidified (pH range 1.5-2.0) using hydrochloric acid.

Extraction of acidic herbicides was carried out using IST ISOLUTE^®^ ENV+ solid-phase extraction cartridges (3 mL, 200 mg, Biotage, Uppsala, Sweden) with custom designed vacuum manifolds. Cartridges were pre-conditioned with ethyl acetate (6 mL), methanol (6 mL) and water (3 mL) and samples loaded to the cartridges at a flow rate of ~ 35 mL min^-1.^ Loaded cartridges were then dried under vacuum. Analytes were eluted under gravity using ethyl acetate (2 x 0.75 mL) followed by acetone (2 x 0.75 mL). The combined eluates were evaporated to ~ 0.5 mL using a Zymark LV evaporator (Caliper Life Sciences Ltd., Runcorn, UK), operating at 40^o^C (water bath temperature) and 15 psi nitrogen pressure.

Methylation was carried out by addition of a solution of diazomethane in diethyl ether (1.0 mL). After a 1 h reaction time, iso-octane (1.0 mL) was added and the derivatised extracts evaporated to ~ 0.1 mL. A further 1.0 mL of iso-octane was added and evaporation carried out to a final volume 0.5 mL. Extracts were spiked with internal standard, 4,4’-dibromooctafluorodiphenyl (0.1 mL of a 1 mg L^-1^ solution in iso-octane) and centrifuged for 4 mins at 2,500 rpm to remove any precipitated material. Supernatants were removed for analysis by GC-MS. The GC-MS analytical performance data are given in Table S2†.

Table S2 Key conditions, together with limits of detection (LoD), used for the gas chromatography-selected ion monitoring mass spectrometry method used for the analysis of twelve acidic herbicides

| **Condition** | **Value** | |
| --- | --- | --- |
| Manufacturer/model | Agilent 6890N/5973N GC-MS | |
| Capillary column | J & W fused silica DB-5MS, 30 m x 0.25 mm, 0.25 µm film thickness | |
| Carrier gas | Helium (30 cm s^-1^ constant flow mode) | |
| Inlet type | Cold on-column (oven track mode) | |
| Injection volume | 1 µL | |
| Oven temperature programme | 55°C (1 min hold), 15°C min^-1^ to 180°C (no hold), 10°C min^-1^ to 250°C (8.7 min hold) | |
| Interface temperature | 280°C | |
| Ionisation mode | Electron impact at 70 eV | |
| Detection mode | Selected ion monitoring | |
| ***Acidic Herbicide*** | ***Ions monitored (quantification & qualification ion)*** | ***LoD in raw surface waters (ng L^-1^)*** |
| Benazolin | 170 & 257 | 10 |
| Clopyralid | 174 7 148 | 10 |
| 2,4-D | 199 & 175 | 7 |
| 2,4-DB | 101 & 162 | 11 |
| Dicamba | 203 & 205 | 7 |
| Dichlorprop | 162 & 248 | 8 |
| Fluroxypyr | 209 & 268 | 11 |
| MCPA | 141 & 214 | 8 |
| MCPB | 101 & 142 | 9 |
| Mecoprop | 228 & 169 | 7 |
| Picloram | 198 & 225 | 12 |
| Triclopyr | 210 & 269 | 8 |

Table S3 Key quality parameters of River Exe water used in the laboratory Chemcatcher^®^ calibration tests

| **Parameter** | **Value** |
| --- | --- |
| pH | 7.8 |
| Dissolved organic carbon (DOC) | 9.4 mg L^-1^ |
| Nitrate | 13.5 mg L^-1^ |
| Ortho-phosphate | 0.03 mg L^-1^ |
| Chloride | 15.0 mg L^-1^ |

**Analytical recoveries of acidic herbicides from 3M Empore™ anion-exchange disks**

In order to assess the suitability of the 3M Empore™ anion-exchange disk for use as the receiving phase in the Chemcatcher^®^, experiments were undertaken to determine the analytical recovery for a range of acidic herbicides monitored routinely by South West Water Ltd. Three aliquots (500 mL) of untreated River Exe water, collected at the inlet to Pynes water treatment works, were spiked (1.0 µg L^-1^ each component) with a mixture of sixteen acidic herbicides (Table S4†). The spiked water samples were extracted on pre-treated Empore™ anion-exchange disks using a filter funnel system connected to a vacuum. The analytes were eluted from the disks using ethyl acetate/acetic acid solution, (10 mL, 9:1 v/v), methylated and analysed by GC-MS (Table S2†) as above. The overall analytical recovery of the herbicides varied (17-98%) and was a function of their physico-chemical properties (Table S5).

The analytical method necessitated the completed removal of any residual acetic acid in the eluates prior the diazomethane derivatisation step. This may cause some evaporative losses for the more volatile compounds. The acidic herbicides with relatively low pK_a_ values (Table S5†) (clopyralid, picloram and, to a lesser extent, dicamba) were poorly eluted from the 3M Empore™ anion-exchange disk. This may be due to the formation of strong ion-pairs with the quaternary ammonium moieties of the extraction phase. Use of alternative stronger elution solvent mixtures (e.g. formic acid) may overcome this problem.

These analytical issues, in conjunction with known application patterns (Table S1†) and detection frequencies within the South West Water Ltd. river catchment, resulted in eight acidic herbicides: 2,4-D, dicamba, dichlorprop, fluroxypyr, MCPA, MCPB, mecoprop and triclopyr, being taken forward into laboratory calibration tests and for investigation in subsequent field trials.

Acidic herbicides can also be analysed using liquid chromatography-mass spectrometry techniques. This analytical method does not require the removal of acetic acid and a methylation step and hence may improve recoveries of these compounds from the 3M Empore™ anion-exchange disk. However, acidic herbicides do exhibit high ionization matrix effects and deuterated internal standards are required for each analyte being quantified.

Table S4 Mean (n = 3) analytical recoveries of twelve acidic herbicides (spiked at 1.0 µg L^-1^) into River Exe water, extracted from the 3M Empore™ anion-exchange disks and analysed using GC-MS. The acidic herbicides in bold were used as analytes in the subsequent laboratory Chemcatcher^®^ uptake rate experiments

| **Acidic herbicide** | **Mean (n = 3) recovery**  **(%)** | **Comment** |
| --- | --- | --- |
| Benazolin | 71 |  |
| Clopyralid | 17 | Very poor elution efficiency |
| **2,4-D** | **75** |  |
| 2,4-DB | 91 |  |
| **Dicamba** | **51** | Poor elution efficiency |
| **Dichlorprop** | **83** |  |
| **Fluroxypyr** | **88** |  |
| **MCPA** | **72** |  |
| **MCPB** | **93** |  |
| **Mecoprop** | **80** |  |
| Picloram | 24 | Very poor elution efficiency |
| **Triclopyr** | **71** |  |

Table S5 Selected physico-chemical properties of the twelve acidic herbicides* used in the analytical performance tests and three pharmaceuticals** found in the catchment. The acidic herbicides in bold type face were used in the laboratory Chemcatcher^®^ uptake rate experiments

| **Compound** | **CAS No.** | **Mono-isotopic molecular weight**  **(g mol^-1^)** | ***pK_a_*** | **Log *K_ow_***  **(pH 7.0 at 20°C)** | **Aqueous solubility**  **(g L^-1^)** |
| --- | --- | --- | --- | --- | --- |
| ***Acidic herbicide*** |  |  |  |  |  |
| Benazolin | 3813-05-6 | 243 | 3.0 | 1.34 | 0.50 |
| Clopyralid | 1702-17-6 | 191 | 2.0 | -2.63 | 143 |
| **2,4-D** | **94-75-7** | **221** | **2.9** | **-0.83** | **23.2** |
| 2,4-DB | 94-82-6 | 248 | 4.1 | 1.35 | 4.39 |
| **Dicamba** | **1918-00-9** | **221** | **1.9** | **-1.88** | **250** |
| **Dichlorprop** | **120-36-5** | **235** | **3.0** | **2.29** | **0.35** |
| **Fluroxypyr** | **69377-81-7** | **255** | **2.9** | **0.04** | **6.50** |
| **MCPA** | **94-74-6** | **201** | **3.7** | **-0.81** | **293** |
| **MCPB** | **94-81-5** | **229** | **4.5** | **1.32** | **4.40** |
| **Mecoprop** | **7085-19-0** | **214** | **3.1** | **-0.19** | **250** |
| Picloram | 1918-02-1 | 240 | 2.3 | -1.92 | 0.56 |
| **Triclopyr** | **55335-06-3** | **257** | **4.0** | **4.62** | **8.10** |
|  |  |  |  |  |  |
| ***Acidic Pharmaceutical*** |  |  |  |  |  |
| Diclofenac | 15307-86-5 | 295 | 4.2 | 4.51 | < 0.01 |
| Ibuprofen | 15687-27-1 | 206 | 4.9 | 3.97 | 0.02 |
| Naproxen | 22204-53-1 | 230 | 4.2 | 3.18 | 0.02 |

*Data obtained from: <http://sitem.herts.ac.uk/aeru/ppdb/en/index.htm> (accessed 15/11/2015)

**Data obtained from: <http://www.drugbank.ca/> (accessed 15/11/2015)


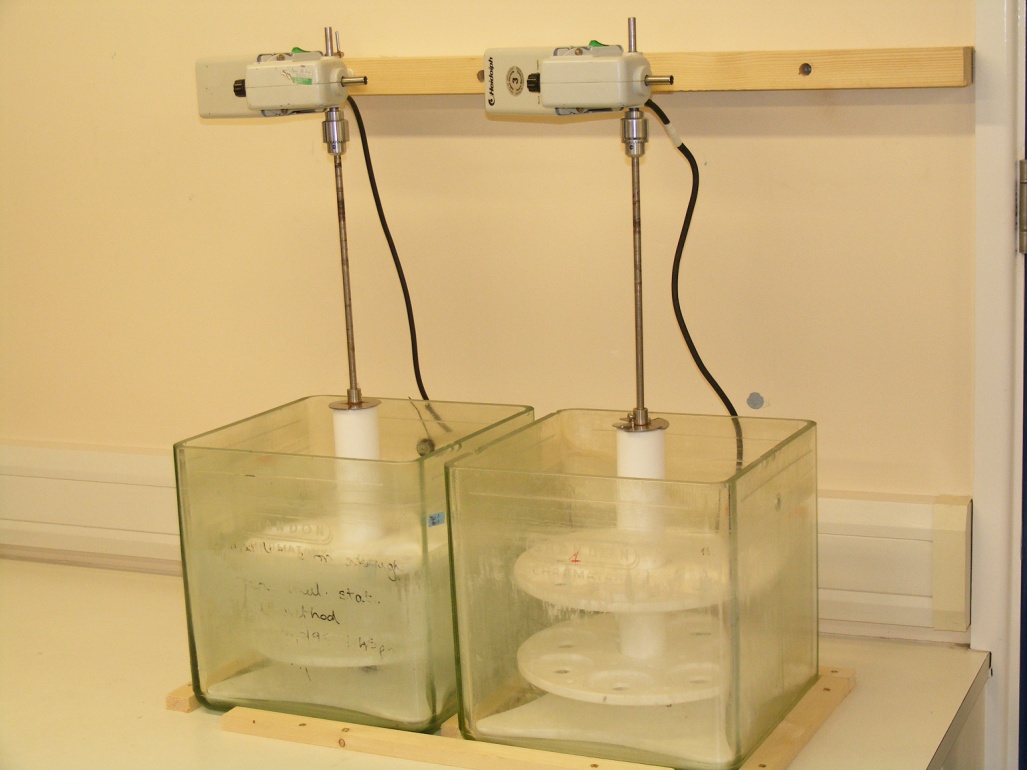


Fig. S2 Glass tank calibration systems fitted with PTFE carousels and overhead stirrers used for the Chemcatcher^®^ laboratory uptake rate experiments for the nine acidic herbicides

**
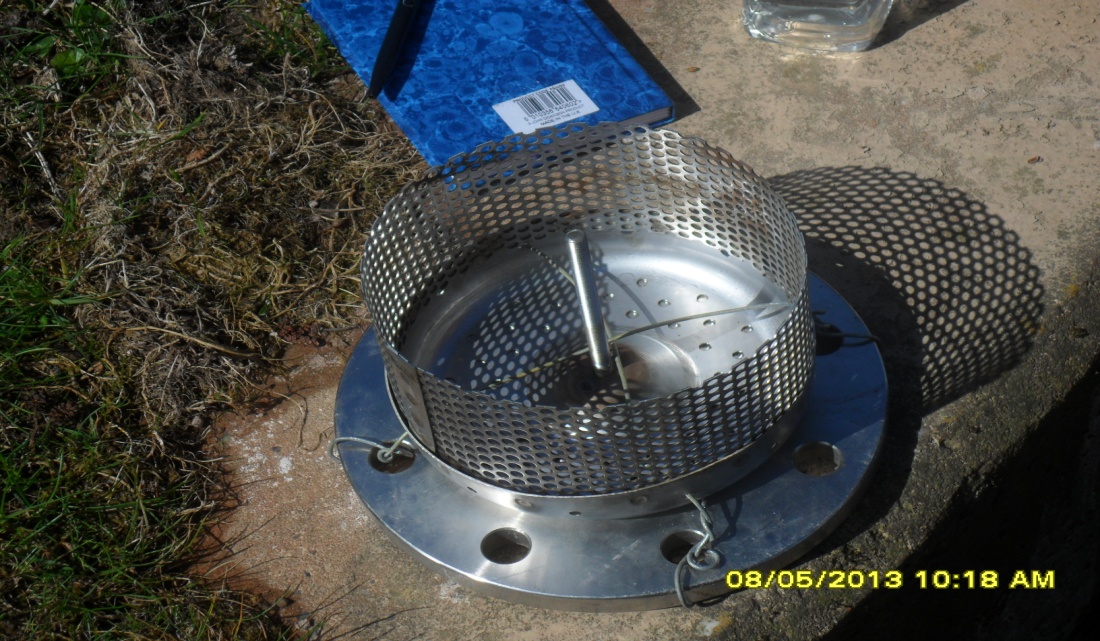
**

1. Stainless steel sampling cage with lid removed.


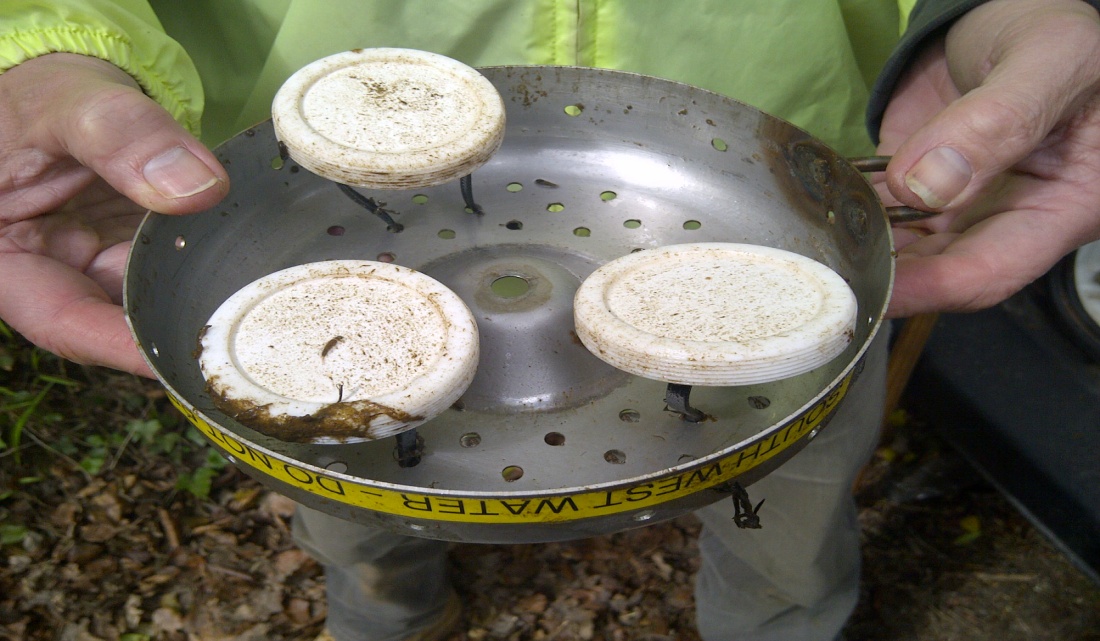


1. Cage lid with three Chemcatcher^®^ passive samplers attached.

**
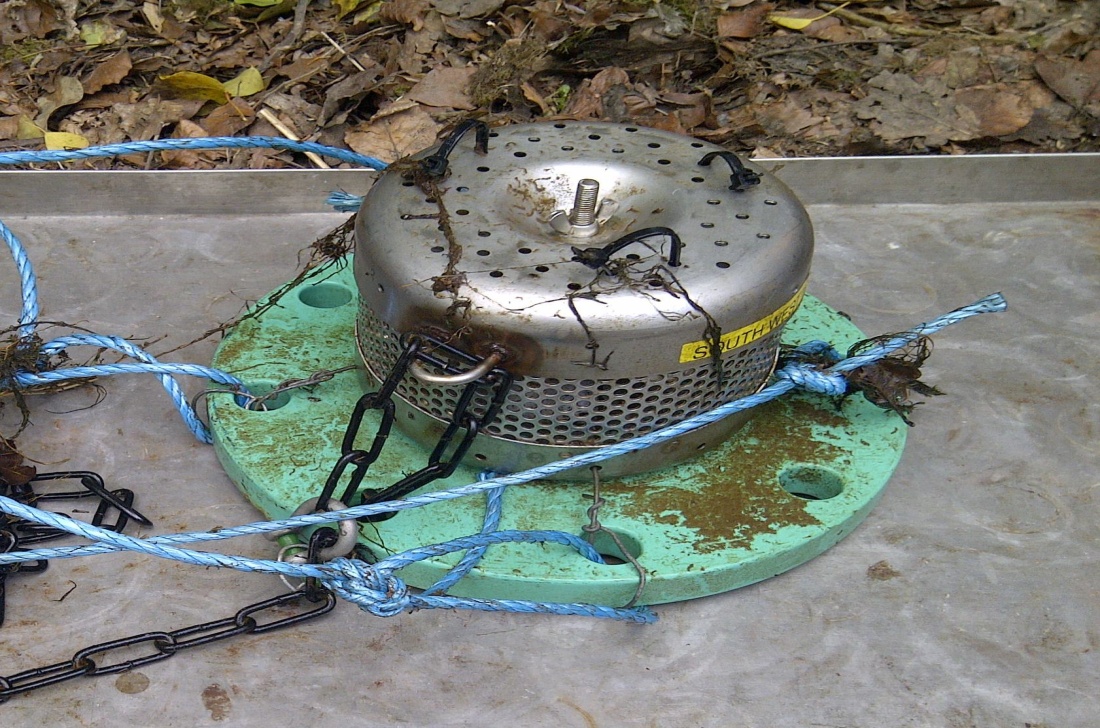
**

(c) Cage after a two week deployment in the River Exe.

Fig. S3 Photographs of stainless steel cages used for the deployment of Chemcatcher^®^ samplers in both field trials

**River Exe catchment and field trial deployment sites**

The River Exe is a relatively large (~ 1500 km^2^), overwhelmingly agricultural catchment. The river rises on the upper slopes of Exmoor and flows southwards for approximately 90 km before entering the English Channel at Exmouth. There are a number of smaller rivers (e.g. River Barle, Batherm, Lowman, Culm, Creddy and Yeo) and streams that enter the main river as it flows south. The upper reaches of the catchment are dominated by hill farming with little associated pesticide application. In the lower catchment (below Bampton) cattle farming begins to predominate resulting in significant use of acidic herbicides on associated grassland. Below Tiverton, land use is mixed, featuring a sizeable element of arable farming, although there are still large acreages of grassland. As a consequence, compounds such as mecoprop and MCPA are detected regularly at the intakes to both Allers and Pynes water treatment works, particularly in the spring and early summer application seasons. The effluent from a large sewage treatment works enters the River Exe below Tiverton.

**
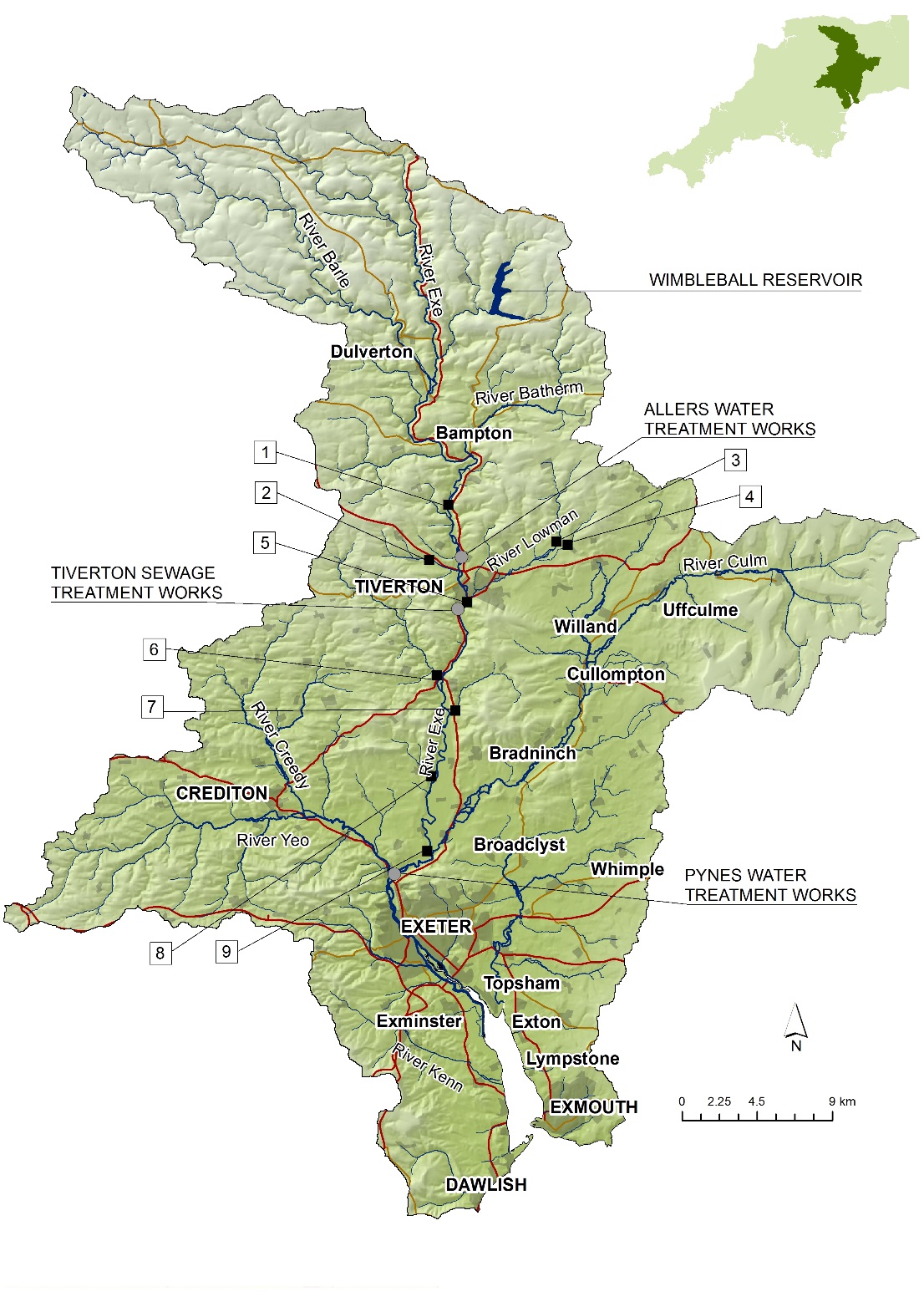
**

Fig. S4 Map of River Exe catchment showing the nine locations for the Chemcatcher^®^ deployments for field trial 2 and the Pynes water treatment works where raw water was collected for the laboratory uptake rate experiments. The sites are numbered in sequence running down the catchment for ease of visualisation. Key to location of sites, together with national grid reference co-ordinates: (1) River Exe at Ironbridge near Stoodleigh (SS94261782), (2) Calverleigh Stream at Lower Farleigh (SS93111452), (3) River Lowman at Uplowman pumping station, (ST00731562) (4) Tributary of River Lowman at Uplowman sewage treatment works (ST01401542), (5) River Lowman at confluence with River Exe (SS95381200), (6) River Dart at Dart Bridge, Bickleigh (SS93570762), (7) River Burn at Burn Mill (SS94670551), (8) Thorverton Stream at Hulk Lane, Thorverton (SS93250158), (9) River Exe at Northbridge intake (SX93009710)

Table S6(a) Mean (n = 32) and variation in water concentrations (µg L^-1^) of acid herbicides in the laboratory calibration tank over a 16 day exposure for Chemcatcher^®^ devices fitted with an overlaying PES membrane. Concentrations at the start of each day and when drained for replenishment after 24 hours were used for all herbicides

| **Compound** | **Mean water concentration of herbicide in tank over deployment (µg L^-1^)** | **Coefficient of variation (%)** | **Range (µg L^-1^)** | **Variation (%) around the mean** |
| --- | --- | --- | --- | --- |
| **2,4-D** | 0.91 | 6.7 | 0.25 | ± 14 |
| **Dicamba** | 1.02 | 4.9 | 0.19 | ± 10 |
| **Dichlorprop** | 0.97 | 5.4 | 0.25 | ± 13 |
| **Fluroxypyr** | 1.04 | 10.0 | 0.50 | ± 24 |
| **MCPA** | 0.94 | 6.5 | 0.24 | ± 13 |
| **MCPB** | 0.93 | 8.2 | 0.26 | ± 14 |
| **Mecoprop** | 0.95 | 6.8 | 0.20 | ± 11 |
| **Triclopyr** | 0.98 | 6.3 | 0.25 | ± 13 |

Table S6(b) Estimated uptake parameters for the eight acidic herbicides used in laboratory calibration tests using Chemcatcher^®^ devices fitted with an overlaying PES membrane. Data were fitted using a standard single exponential model using GenStat 15 (VSN International Ltd.). In Genstat the curve is fitted as A+B(R^X^). The tank test was operated for 16 days. Acidic herbicides were not detectable in the laboratory blank samplers

| **Acidic herbicide** | **R (s.e.)** | **B (s.e.)**  **(µg L^-1^)** | **A (s.e.)**  **(µg L^-1^)** | ***k_e_***  **(h^-1^)** | ***t_(0.5)_***  **(days)** |
| --- | --- | --- | --- | --- | --- |
| **2,4-D** | 0.99828  (0.00132) | -1.91  (1.050) | 1.93  (1.08) | -0.00172 | 17.0 |
| **Dicamba** | 0.99818  (0.00149) | -1.301  (0.748) | 1.330  (0.772) | -0.00182 | 16.0 |
| **Dichlorprop** | 0.99541  (0.00135) | -1.371  (0.170) | 1.385  (0.196) | -0.00460 | 6.3 |
| **Fluroxypyr** | 0.99553  (0.00193) | -0.81  (0.149) | 0.823  (0.171) | -0.00448 | 6.4 |
| **MCPA** | 0.99950  (0.00157) | -5.4  (15.50) | 5.4  (15.5) | -0.00050 | 58.0 |
| **MCPB** | 0.99728  (0.00221) | -1.118  (0.535) | 1.173  (0.572) | -0.00272 | 11.0 |
| **Mecoprop** | 0.99517  (0.00148) | -1.334  (0.165) | 1.360  (0.192) | -0.00484 | 6.0 |
| **Triclopyr** | 0.99659  (0.00173) | -0.854  (0.223) | 0.874  (0.245) | -0.00342 | 8.5 |

**Key:**

s.e. = standard error; R = exp(*k_e_*); B = lower intercept; A = upper asymptote;

*k_e_ =* first order rate constant; *t_(0.5)_=* half-time to equilibrium (maximum).

Fitting an exponential function to data sets where there is little information about the curvature or upper asymptote is problematic. The estimates of the parameters A and B (upper limit, and lower asymptote, respectively) are based on a few points and tend to be correlated, and hence unreliable since the fit would not be changed if A were raised and B lowered by a matching value. For some compounds (2,4-D, dicamba, and MCPA) fitting a straight line by linear regression gave a fit that was as good as that obtained by asymptotic regression as judged by the pattern of residuals and r^2^ (the proportion of variation accounted for by the regression). For other compounds (dichlorprop, fluroxypyr, and mecoprop) the asymptotic regression gave better fits in terms of bias in the residuals and r^2^. For some compounds it would be possible to use some time points above the t_(0.5)_ value. This was established by incrementing the number of time points used in the linear regression in a stepwise manner and examining the pattern of residuals, the slope value, and the value of the intercept term. If points above the linear portion are added, the intercept value becomes significantly different from zero, and bias is seen in the pattern of residuals. On this basis it would be possible to extend the range of dichlorprop (from six to eleven days), fluroxypyr (from six to 10 days), mecoprop (from six to eleven days), triclopyr (from eight to eleven days) without any marked effect on the value of the slope. For dicamba it would not be possible to extend the exposure time used.

Table S6(c) Summary of linear regression parameters obtained from fitting a straight line relationship using observations up to the estimated t_(0.5)_ value. The uptake rate constant (*R_s_*) was calculated using the slope of the regression line, and the average concentration in the tank over the whole calibration period according to equation 1. The uncertainty for *R_s_* was calculated using standard methods for combining errors.

| **Acidic herbicide** | **Number of exposure times used (n)** | **Slope (s.e.)**  **(µg h^-1^)** | **Intercept** | **Probability**  **that intercept is zero** | ***R_s_***  **(± Δ)**  **(L day^-1^)** |
| --- | --- | --- | --- | --- | --- |
| **2,4-D** | 14 | 0.002411  (0.00017) | 0.065 | 0.071 | 0.064 (0.009) |
| **Dicamba** | 12 | 0.001855  (0.000199) | 0.048 | 0.130 | 0.044 (0.007) |
| **Dichlorprop** | 8 | 0.0045  (0.000708) | 0.050 | 0.417 | 0.112 (0.024) |
| **Fluroxypyr** | 8 | 0.003137  (0.000469) | 0.011 | 0.776 | 0.073 (0.018) |
| **MCPA** | 14 | 0.002427  (0.000193) | 0.080 | 0.053 | 0.062 (0.009) |
| **MCPB** | 11 | 0.002377  (0.00032) | 0.073 | 0.108 | 0.062 (0.013) |
| **Mecoprop** | 8 | 0.004464  (0.000696) | 0.071 | 0.258 | 0.113 (0.025) |
| **Triclopyr** | 10 | 0.002175  (0.000251) | 0.039 | 0.190 | 0.053 (0.010) |

**Key:**

Δ = uncertainty

s.e. = standard error


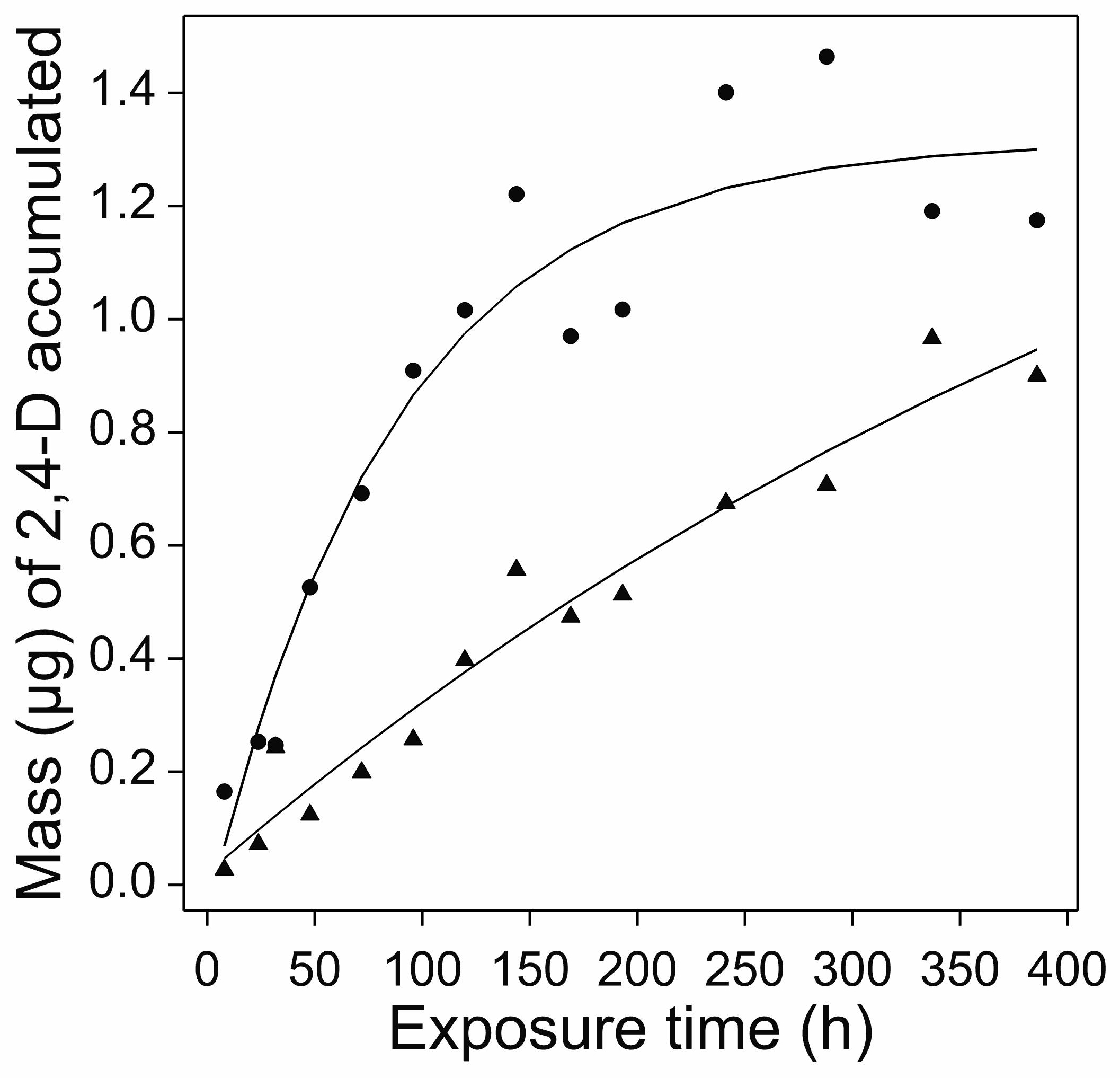


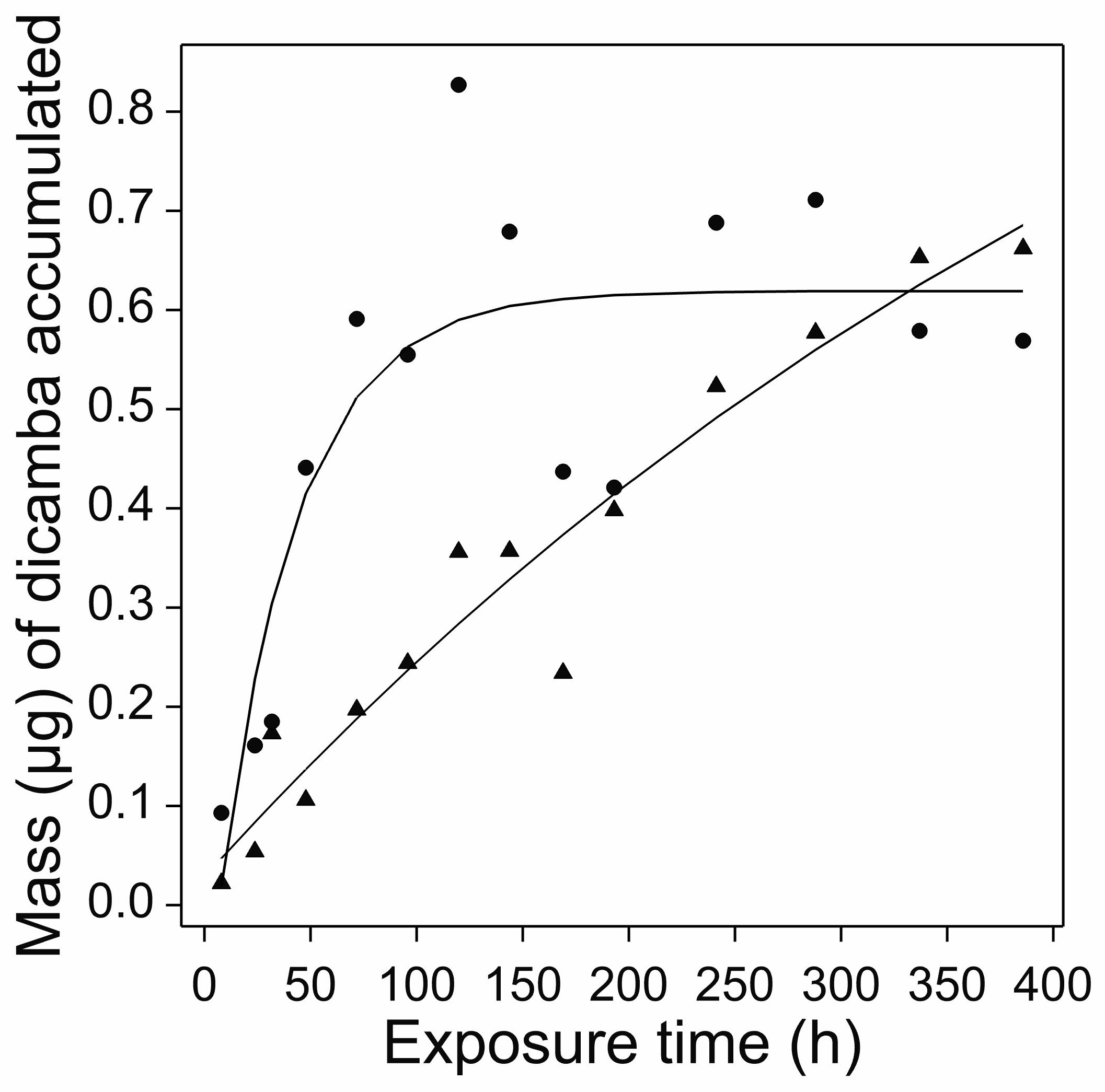


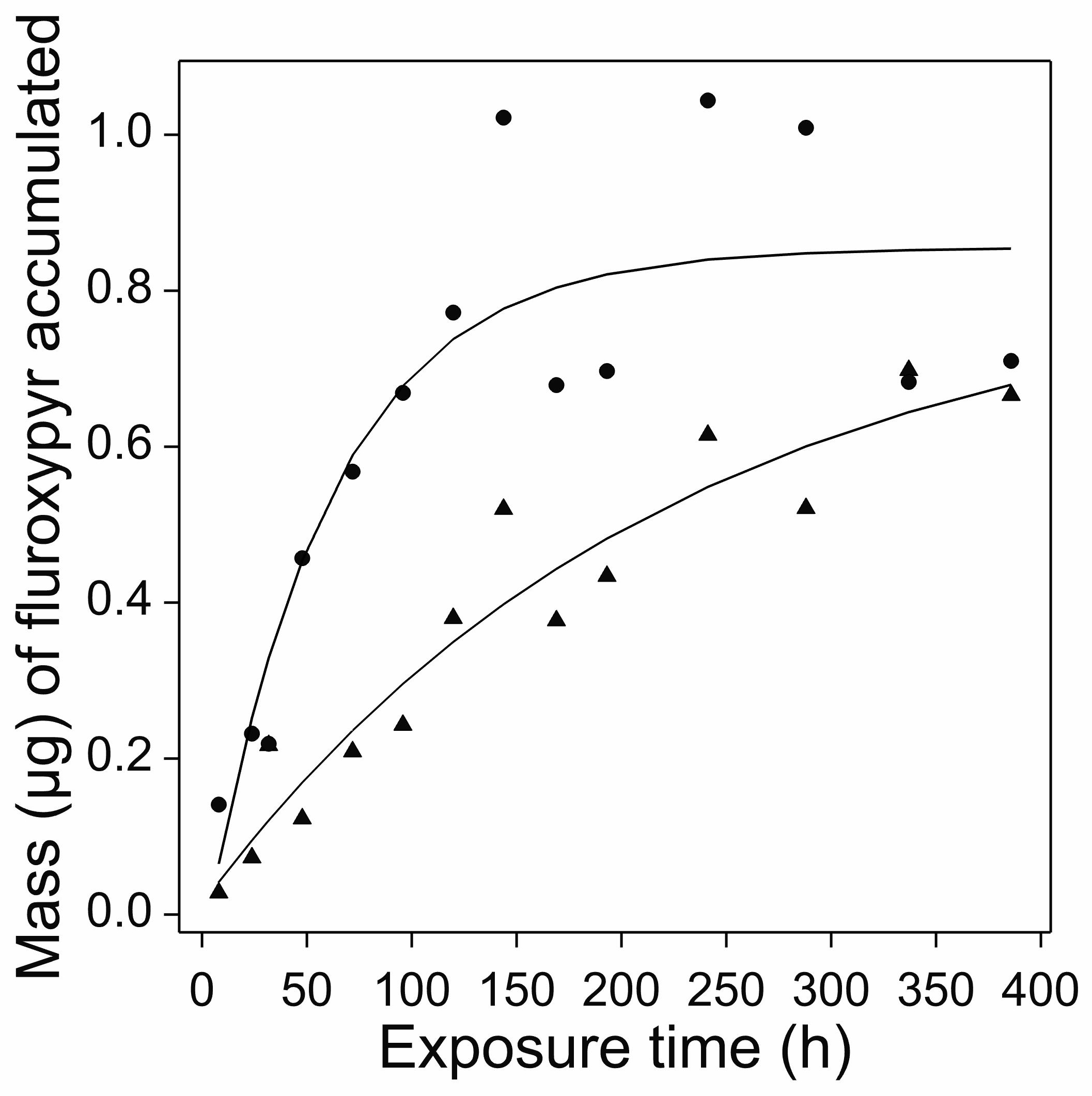


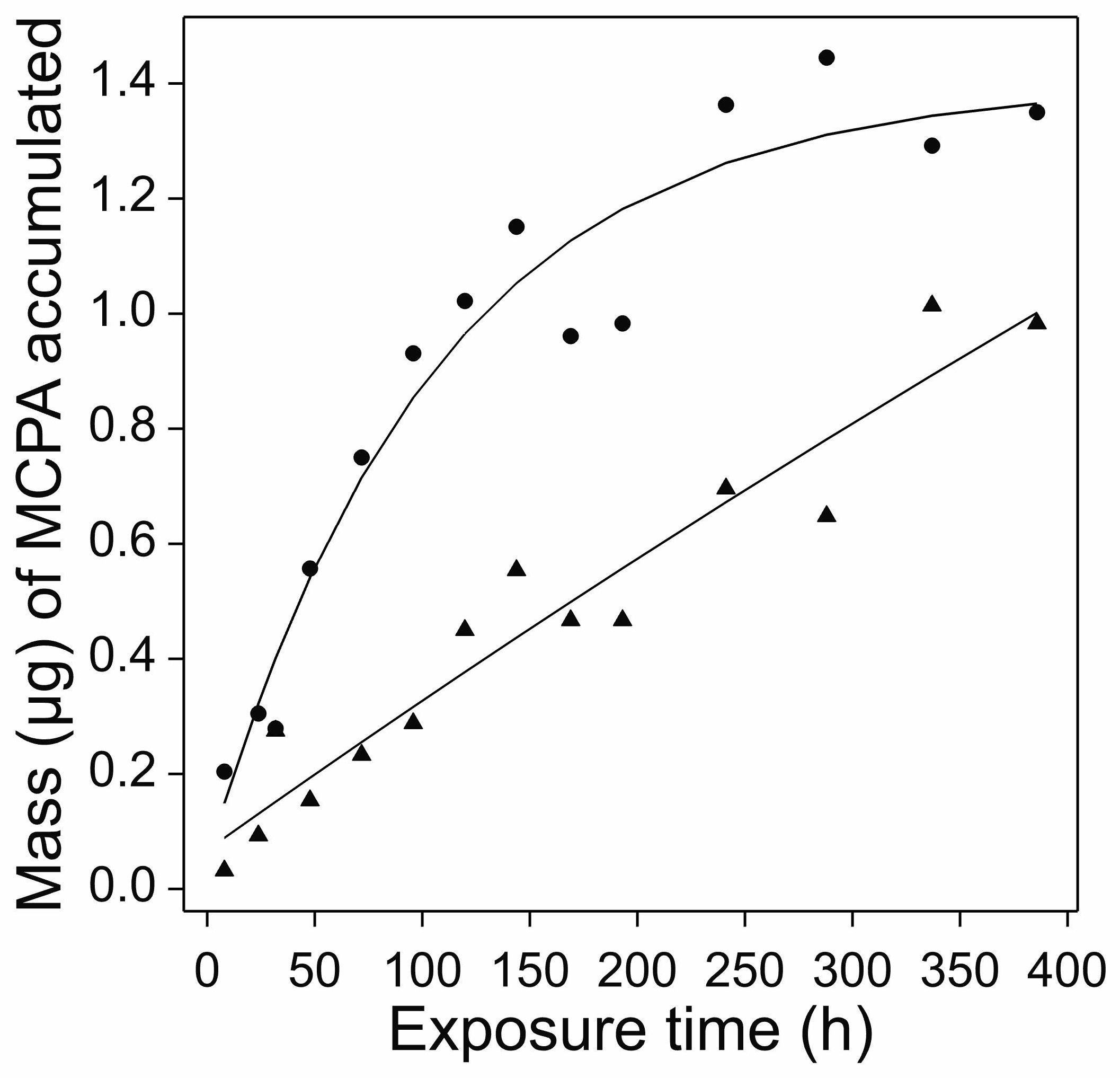


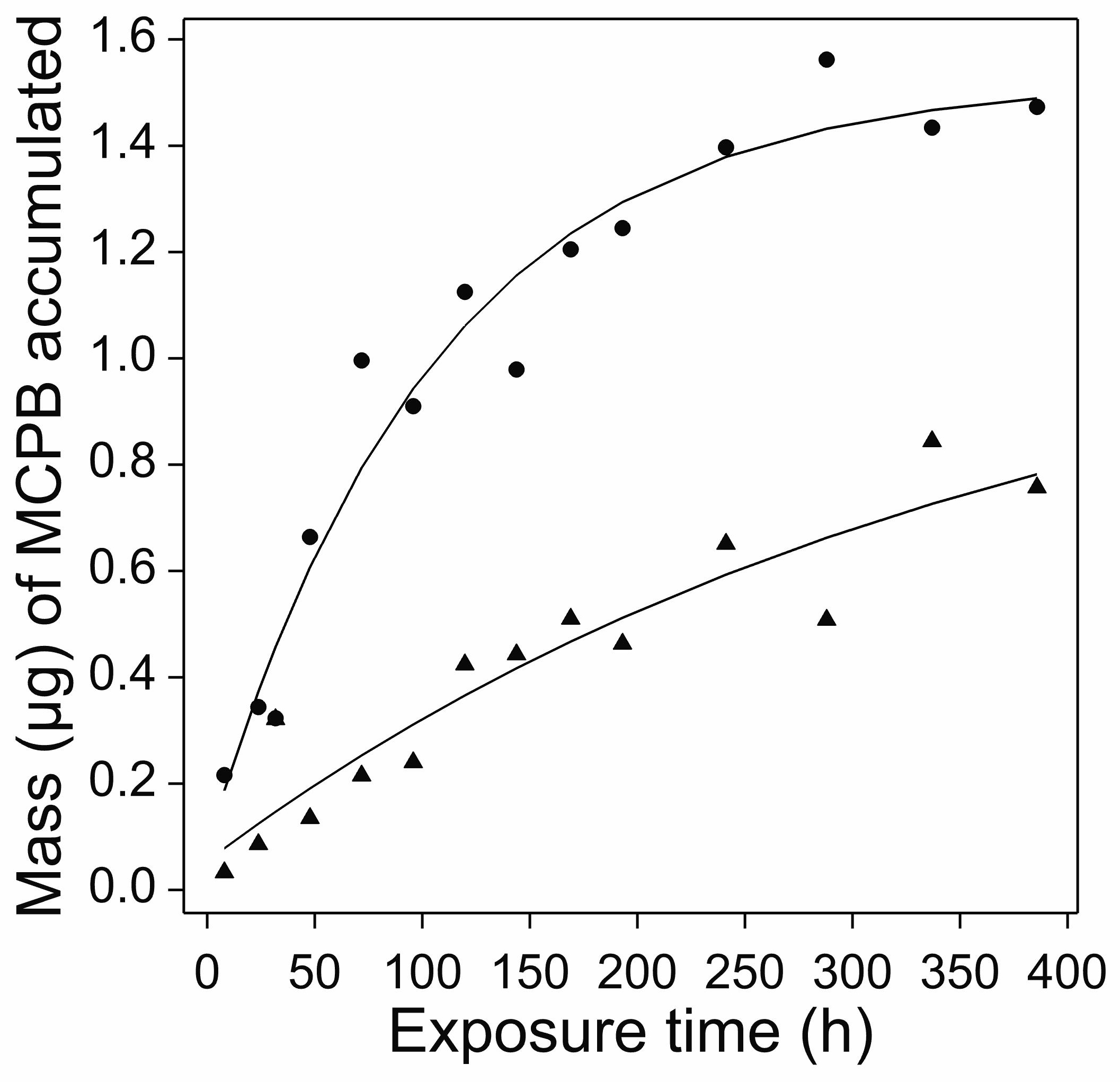


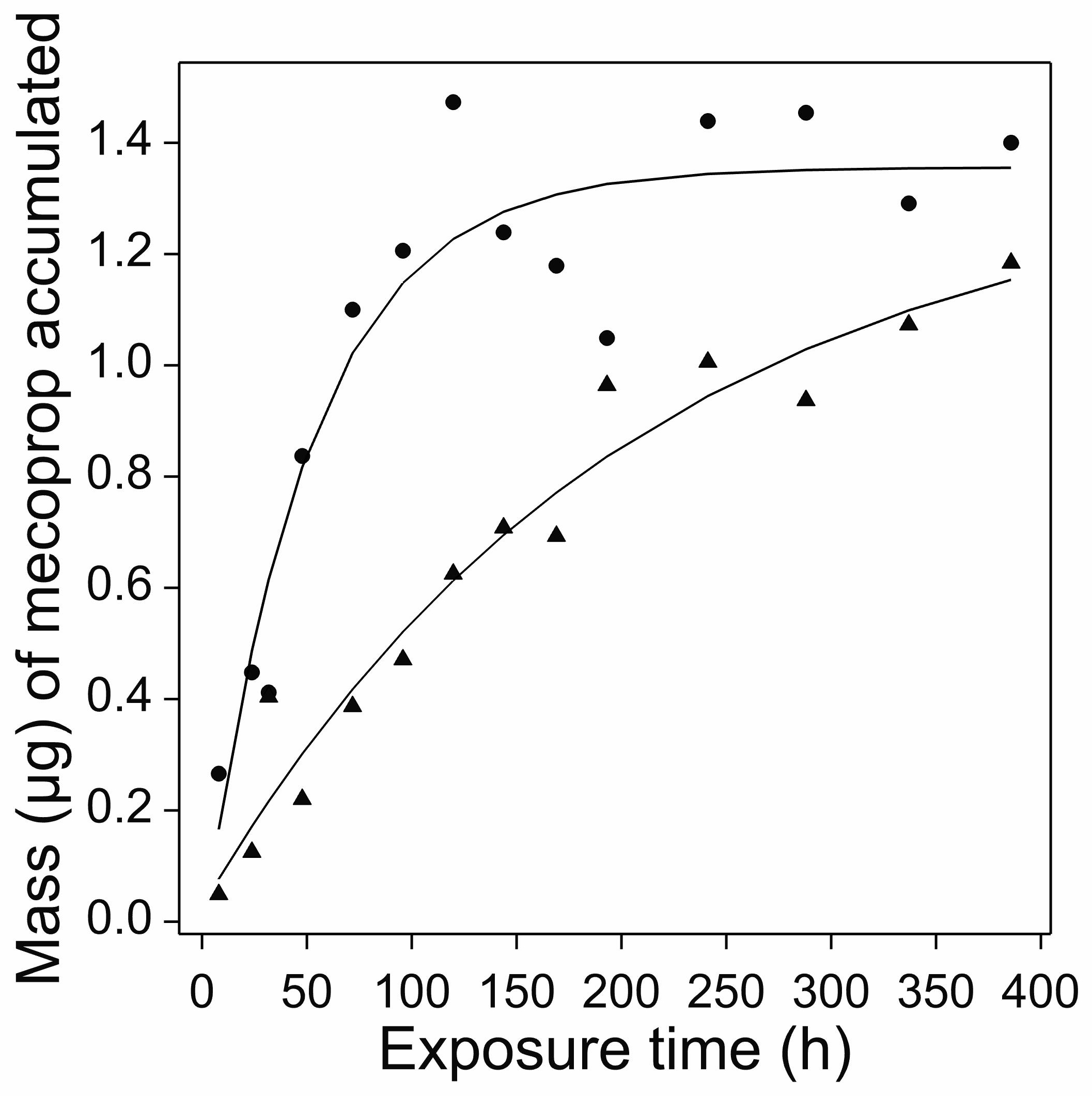


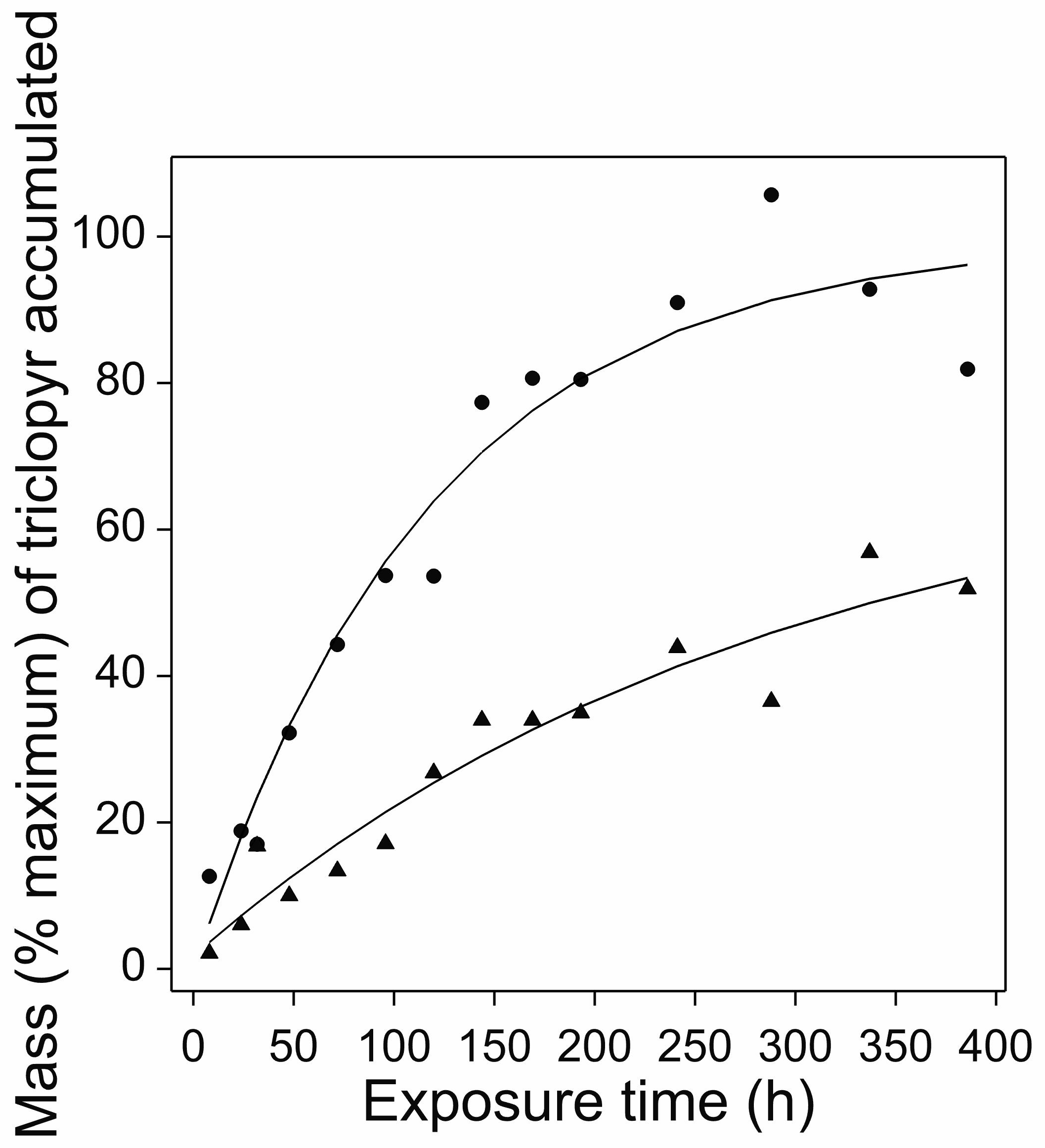


Fig. S5. Uptake curves for the seven other acidic herbicides tested in the laboratory calibration tests using Chemcatcher^®^ devices fitted with an overlaying PES membrane (▲) no PES membrane (⚫). Curves were fitted using the standard exponential function in the non-linear regression routine of GenStat 15 (VSN International Ltd.). With the exception of triclopyr, there was no significant differences (paired sample t-tests) between the mean concentrations of herbicides in the tanks used in parallel for calibrating passive samplers with and without a diffusion limiting membrane. Hence, it was possible to plot the curves on common axes. In order to bring the uptake curves for triclopyr onto the same scale the masses accumulated were expressed as a percentage of the estimated maxima for the two tanks.

Table S7(a) Aqueous concentration and estimated time-weighted average (TWA) concentration measured by the Chemcatcher^®^ (CC) (n = 3) for eight acidic herbicides, together with the river water properties in field trial 1 at site 1 (River Barle at Pixton Hill above Brushford sewage treatment works (national grid reference co-ordinates SS92482625)). The concentration of acidic herbicides found in the Chemcatcher^®^ field blanks was below the detection limit of the analytical method. Bold type face shows elevated concentrations

| **Acidic herbicide** | Concentration (ng L^-1^) in spot water samples | | | | | | | CC 1  TWA*  (ng L^-1^) | CC 2  TWA*  (ng L^-1^) | CC 3  TWA*  (ng L^-1^) | Average  TWA*  (ng L^-1^) |
| --- | --- | --- | --- | --- | --- | --- | --- | --- | --- | --- | --- |
|  | Day  0 | Day  2 | Day  5 | Day  7 | Day  9 | Day  12 | Day  14 |  |  |  |  |
| 2,4-D | <7 | <7 | <7 | <7 | <7 | <7 | <7 | <1.12 | <1.12 | <1.12 | <1.12 |
| Dicamba | <7 | <7 | <7 | <7 | <7 | <7 | <7 | <1.62 | <1.62 | <1.62 | <1.62 |
| Dichlorprop | <8 | <8 | <8 | <8 | <8 | <8 | <8 | <0.64 | <0.64 | <0.64 | <0.64 |
| Fluroxypyr | <11 | <11 | <11 | <11 | <11 | <11 | <11 | <0.98 | <0.98 | <0.98 | <0.98 |
| MCPA | <8 | <8 | **77** | <8 | <8 | <8 | <8 | <1.15 | 1.84 | 1.73 | 1.79 |
| MCPB | <9 | <9 | <9 | <9 | <9 | <9 | <9 | <1.15 | <1.15 | <1.15 | <1.15 |
| Mecoprop | <7 | <7 | **13** | <7 | <7 | <7 | <7 | <0.70 | <0.76 | <0.82 | <0.82 |
| Triclopyr | <8 | <8 | <8 | <8 | <8 | <8 | <8 | 2.29 | 1.48 | <1.35 | 1.89 |
|  |  |  |  |  |  |  |  |  |  |  |  |
| **Water properties** |  |  |  |  |  |  |  |  |  |  |  |
| Temp  (°C) | 13.0 | 13.0 | 11.0 | 8.5 | 10.8 | 12.9 | 11.7 |  |  |  |  |
| pH | 6.75 | 6.94 | 8.16 | 7.30 | 7.94 | 7.97 | 7.27 |  |  |  |  |
| Flow  (m s^-1^) | 0.20 | n/a | 0.35 | 1.00 | 0.45 | 0.45 | 0.45 |  |  |  |  |
| Nitrate  (mg L^-1^) | 1.7 | 2.1 | 1.7 | 1.6 | 2.0 | 2.1 | 1.6 |  |  |  |  |
| DOC  (mg L^-1^) | 2.0 | 2.7 | 2.3 | 6.7 | 1.3 | 1.1 | 1.0 |  |  |  |  |

n/a = measurement not available

*Estimated TWA concentration assuming the Chemcatcher^®^ samplers (CC) were in the time integrative mode for all nine compounds over the 14-day deployment.

Table S7(b) Aqueous concentration and estimated time-weighted average (TWA) concentration measured by the Chemcatcher^®^ (CC) (n = 3) for eight acidic herbicides, together with the river water properties in field trial 1 at site 2 (River Exe at Exebridge pumping station (national grid reference co-ordinates SS93012447)). The concentration of acidic herbicides found in the Chemcatcher^®^ field blanks was below the detection limit of the analytical method

| **Acidic herbicide** | Concentration (ng L^-1^) in spot water samples | | | | | | | CC 1  TWA*  (ng L^-1^) | CC 2  TWA*  (ng L^-1^) | CC 3  TWA*  (ng L^-1^) | Average  TWA*  (ng L^-1^) |
| --- | --- | --- | --- | --- | --- | --- | --- | --- | --- | --- | --- |
|  | Day  0 | Day  2 | Day  5 | Day  7 | Day  9 | Day  12 | Day  14 |  |  |  |  |
| 2,4-D | <7 | <7 | <7 | <7 | <7 | <7 | <7 | <1.12 | <1.12 | <1.12 | <1.12 |
| Dicamba | <7 | <7 | <7 | <7 | <7 | <7 | <7 | <1.62 | <1.62 | <1.62 | <1.62 |
| Dichlorprop | <8 | <8 | <8 | <8 | <8 | <8 | <8 | <0.64 | <0.64 | <0.64 | <0.64 |
| Fluroxypyr | <11 | <11 | <11 | <11 | <11 | <11 | <11 | <0.98 | <0.98 | <0.98 | <0.98 |
| MCPA | <8 | <8 | <8 | <8 | <8 | <8 | <8 | **1.20** | **1.50** | **1.50** | **1.38** |
| MCPB | <9 | <9 | <9 | <9 | <9 | <9 | <9 | <1.15 | <1.15 | <1.15 | <1.15 |
| Mecoprop | <7 | <7 | <7 | <7 | <7 | <7 | <7 | <0.63 | <0.63 | <0.63 | <0.63 |
| Triclopyr | <8 | <8 | <8 | <8 | <8 | <8 | <8 | **1.75** | **1.62** | **2.02** | **1.80** |
|  |  |  |  |  |  |  |  |  |  |  |  |
| **Water properties** |  |  |  |  |  |  |  |  |  |  |  |
| Temp  (°C) | 13.0 | 13.0 | 10.8 | 9.6 | 10.9 | 12.6 | 11.5 |  |  |  |  |
| pH | 7.33 | 7.42 | 8.65 | 7.41 | 7.81 | 7.92 | 7.52 |  |  |  |  |
| Flow  (m s^-1^) | n/a | n/a | n/a | n/a | n/a | n/a | n/a |  |  |  |  |
| Nitrate  (mg L^-1^) | 2.9 | 4.8 | 3.2 | 1.6 | 3.1 | 3.1 | 2.6 |  |  |  |  |
| DOC  (mg L^-1^) | 1.9 | 2.2 | 1.7 | 6.2 | 1.3 | 1.2 | 1.1 |  |  |  |  |

n/a = measurement not available

*Estimated TWA concentration assuming the Chemcatcher^®^ samplers (CC) were in the time integrative mode for all nine compounds over the 14-day deployment.

Table S7(c) Aqueous concentration and estimated time-weighted average (TWA) concentration measured by the Chemcatcher^®^ (CC) (n = 3) for eight acidic herbicides, together with the river water properties in field trial 1 at site 3 (River Exe at Ironbridge near Stoodleigh (national grid reference co-ordinates SS94261782)). The concentration of acidic herbicides found in the Chemcatcher^®^ field blanks was below the detection limit of the analytical method. Bold type face shows elevated concentrations

| **Acidic herbicide** | Concentration (ng L^-1^) in spot water samples | | | | | | | CC 1  TWA*  (ng L^-1^) | CC 2  TWA*  (ng L^-1^) | CC 3  TWA*  (ng L^-1^) | Average  TWA*  (ng L^-1^) |
| --- | --- | --- | --- | --- | --- | --- | --- | --- | --- | --- | --- |
|  | Day  0 | Day  2 | Day  5 | Day  7 | Day  9 | Day  12 | Day  14 |  |  |  |  |
| 2,4-D | <7 | <7 | <7 | <7 | <7 | <7 | <7 | **4.58** | **3.13** | **3.68** | **3.79** |
| Dicamba | <7 | <7 | <7 | <7 | <7 | <7 | <7 | <1.62 | <1.62 | <1.62 | <1.62 |
| Dichlorprop | <8 | <8 | <8 | <8 | <8 | <8 | <8 | <0.64 | <0.64 | <0.64 | <0.64 |
| Fluroxypyr | <11 | <11 | <11 | <11 | <11 | <11 | <11 | <0.98 | <0.98 | <0.98 | <0.98 |
| MCPA | <8 | <8 | <8 | **20** | <8 | <8 | <8 | **9.91** | **11.1** | **12.44** | **11.14** |
| MCPB | <9 | <9 | <9 | <9 | <9 | <9 | <9 | <1.15 | <1.15 | <1.15 | <1.15 |
| Mecoprop | <7 | <7 | <7 | <7 | <7 | <7 | <7 | **0.88** | **0.95** | **1.01** | **0.95** |
| Triclopyr | <8 | <8 | <8 | <8 | <8 | <8 | <8 | **3.37** | **2.56** | **3.50** | **3.14** |
|  |  |  |  |  |  |  |  |  |  |  |  |
| **Water properties** |  |  |  |  |  |  |  |  |  |  |  |
| Temp  (°C) | 14.8 | 13.5 | 10.7 | 8.9 | 10.6 | 13.2 | 12.1 |  |  |  |  |
| pH | 7.53 | 7.67 | 8.30 | 7.46 | 7.82 | 8.10 | 7.52 |  |  |  |  |
| Flow  (m s^-1^) | 0.35 | n/a | 0.60 | 0.80 | 0.60 | 0.50 | 0.55 |  |  |  |  |
| Nitrate  (mg L^-1^) | 3.0 | 5.0 | 3.4 | 1.6 | 3.9 | 3.5 | 2.6 |  |  |  |  |
| DOC  (mg L^-1^) | 2.2 | 2.0 | 3.0 | 6.0 | 1.7 | 1.7 | 1.4 |  |  |  |  |

n/a = measurement not available

*Estimated TWA concentration assuming the Chemcatcher^®^ samplers (CC) were in the time integrative mode for all nine compounds over the 14-day deployment.

Table S7(d) Aqueous concentration and estimated time-weighted average (TWA) concentration measured by the Chemcatcher^®^ (CC) (n = 3) for eight acidic herbicides, together with the river water properties in field trial 1 at site 4 (River Lowman at confluence with River Exe (national grid reference co-ordinates SS95381200)). The concentration of acidic herbicides found in the Chemcatcher^®^ field blanks was below the detection limit of the analytical method. Bold type face shows elevated concentrations

| **Acidic herbicide** | Concentration (ng L^-1^) in spot water samples | | | | | | | CC 1  TWA*  (ng L^-1^) | CC 2  TWA*  (ng L^-1^) | CC 3  TWA*  (ng L^-1^) | Average  TWA*  (ng L^-1^) |
| --- | --- | --- | --- | --- | --- | --- | --- | --- | --- | --- | --- |
|  | Day  0 | Day  2 | Day  5 | Day  7 | Day  9 | Day  12 | Day  14 |  |  |  |  |
| 2,4-D | <7 | <7 | <7 | <7 | <7 | <7 | <7 | **4.13** | **3.91** | **3.91** | **3.98** |
| Dicamba | <7 | <7 | <7 | <7 | <7 | <7 | <7 | **5.68** | **6.49** | **4.38** | **5.52** |
| Dichlorprop | <8 | <8 | <8 | <8 | <8 | <8 | <8 | <0.64 | <0.64 | <0.64 | <0.64 |
| Fluroxypyr | <11 | <11 | <11 | <11 | <11 | <11 | <11 | **6.07** | **5.97** | **5.38** | **5.81** |
| MCPA | <8 | <8 | <8 | **35** | <8 | **19** | <8 | **11.64** | **11.98** | **13.13** | **12.25** |
| MCPB | <9 | <9 | <9 | <9 | <9 | <9 | <9 | <1.15 | <1.15 | <1.15 | <1.15 |
| Mecoprop | **868** | **25** | <7 | **53** | **36** | **21** | **144** | **68.46** | **70.99** | **65.17** | **68.20** |
| Triclopyr | <8 | <8 | <8 | **17** | <8 | **10** | <8 | **14.42** | **14.15** | **14.69** | **14.42** |
|  |  |  |  |  |  |  |  |  |  |  |  |
| **Water properties** |  |  |  |  |  |  |  |  |  |  |  |
| Temp  (°C) | 14.0 | 14.5 | 11.1 | 9.3 | 10.1 | 12.2 | 14.1 |  |  |  |  |
| pH | 8.20 | 8.24 | 8.43 | 8.02 | 8.66 | 8.36 | 8.17 |  |  |  |  |
| Flow  (m s^-1^) | 0.25 | n/a | 0.20 | 0.33 | 0.25 | 0.20 | 0.2 |  |  |  |  |
| Nitrate  (mg L^-1^) | 24.4 | 27.1 | 25.2 | 19.9 | 24.8 | 24.7 | 24.2 |  |  |  |  |
| DOC  (mg L^-1^) | 3.7 | 2.5 | 2.3 | 7.5 | 2.5 | 2.6 | 2.3 |  |  |  |  |

n/a = measurement not available

*Estimated TWA concentration assuming the Chemcatcher^®^ samplers (CC) were in the time integrative mode for all nine compounds over the 14-day deployment.

Table S7(e) Aqueous concentration and estimated time-weighted average (TWA) concentration measured by the Chemcatcher^®^ (CC) (n = 3) for eight acidic herbicides, together with the river water properties in field trial 1 at site 5 (River Exe upstream of Tiverton sewage treatment works (national grid reference co-ordinates SS95191104)). The concentration of acidic herbicides found in the Chemcatcher^®^ field blanks was below the detection limit of the analytical method. Bold type face shows elevated concentrations

| **Acidic herbicide** | Concentration (ng L^-1^) in spot water samples | | | | | | | CC 1  TWA*  (ng L^-1^) | CC 2  TWA*  (ng L^-1^) | CC 3  TWA*  (ng L^-1^) | Average  TWA*  (ng L^-1^) |
| --- | --- | --- | --- | --- | --- | --- | --- | --- | --- | --- | --- |
|  | Day  0 | Day  2 | Day  5 | Day  7 | Day  9 | Day  12 | Day  14 |  |  |  |  |
| 2,4-D | <7 | <7 | <7 | **24** | <7 | <7 | <7 | **2.90** | **4.02** | **3.46** | **3.46** |
| Dicamba | <7 | <7 | <7 | <7 | <7 | <7 | <7 | <1.62 | <1.62 | <1.62 | <1.62 |
| Dichlorprop | <8 | <8 | <8 | <8 | <8 | <8 | <8 | <0.64 | <0.64 | <0.64 | <0.64 |
| Fluroxypyr | <11 | <11 | <11 | <11 | <11 | <11 | <11 | <0.98 | <0.98 | **2.54** | **2.54** |
| MCPA | <8 | <8 | <8 | **36** | <8 | <8 | <8 | **8.29** | **8.06** | **8.41** | **8.26** |
| MCPB | <9 | <9 | <9 | <9 | <9 | <9 | <9 | <1.15 | <1.15 | <1.15 | <1.15 |
| Mecoprop | **45** | <7 | <7 | <7 | <7 | <7 | **10** | **5.94** | **6.64** | **6.13** | **6.24** |
| Triclopyr | <8 | <8 | <8 | <8 | <8 | <8 | <8 | **6.87** | **5.26** | **4.04** | **5.39** |
|  |  |  |  |  |  |  |  |  |  |  |  |
| **Water properties** |  |  |  |  |  |  |  |  |  |  |  |
| Temp  (°C) | 15.0 | 14.0 | 10.7 | 8.7 | 9.7 | 12.5 | 13.5 |  |  |  |  |
| pH | 8.15 | 8.05 | 8.29 | 7.60 | 7.89 | 8.01 | 7.99 |  |  |  |  |
| Flow  (m s^-1^) | 0.50 | n/a | 0.60 | 1.75 | 0.35 | 0.60 | 0.65 |  |  |  |  |
| Nitrate  (mg L^-1^) | 7.4 | 7.2 | 5.9 | 2.3 | 5.6 | 5.8 | 5.0 |  |  |  |  |
| DOC  (mg L^-1^) | 2.4 | 1.9 | 2.8 | 6.7 | 2.0 | 2.5 | 1.7 |  |  |  |  |

n/a = measurement not available

*Estimated TWA concentration assuming the Chemcatcher^®^ samplers (CC) were in the time integrative mode for all nine compounds over the 14-day deployment.

Table S7(f) Aqueous concentration and estimated time-weighted average (TWA) concentration measured by the Chemcatcher^®^ (CC) (n = 3) for eight acidic herbicides, together with the river water properties in field trial 1 at site 6 (River Exe downstream of Tiverton sewage treatment works (national grid reference co-ordinates SS95381018)). The concentration of acidic herbicides found in the Chemcatcher^®^ field blanks was below the detection limit of the analytical method. Bold type face shows elevated concentrations

| **Acidic herbicide** | Concentration (ng L^-1^) in spot water samples | | | | | | | CC 1  TWA*  (ng L^-1^) | CC 2  TWA*  (ng L^-1^) | CC 3  TWA*  (ng L^-1^) | Average  TWA*  (ng L^-1^) |
| --- | --- | --- | --- | --- | --- | --- | --- | --- | --- | --- | --- |
|  | Day  0 | Day  2 | Day  5 | Day  7 | Day  9 | Day  12 | Day  14 |  |  |  |  |
| 2,4-D | <7 | <7 | <7 | **20** | <7 | <7 | <7 | **3.79** | **3.57** | **3.24** | **3.53** |
| Dicamba | <7 | <7 | <7 | <7 | **14** | <7 | <7 | <1.62 | <1.62 | <1.62 | <1.62 |
| Dichlorprop | <8 | <8 | <8 | <8 | <8 | <8 | <8 | <0.64 | <0.64 | <0.64 | <0.64 |
| Fluroxypyr | <11 | <11 | <11 | <11 | <11 | <11 | <11 | 1.37 | <0.98 | 1.17 | 1.27 |
| MCPA | <8 | <8 | <8 | **36** | <8 | <8 | <8 | **11.52** | **7.37** | **9.79** | **9.56** |
| MCPB | <9 | <9 | <9 | <9 | <9 | <9 | <9 | <1.15 | <1.15 | <1.15 | <1.15 |
| Mecoprop | <7 | <7 | <7 | <7 | **8** | <7 | **15** | **8.91** | **6.70** | **6.95** | **7.52** |
| Triclopyr | <8 | <8 | <8 | <8 | <8 | <8 | <8 | **4.72** | **3.50** | **4.04** | **4.09** |
|  |  |  |  |  |  |  |  |  |  |  |  |
| **Water properties** |  |  |  |  |  |  |  |  |  |  |  |
| Temp  (°C) | 14.8 | 13.5 | 9.3 | 9.4 | 10.0 | 12.7 | 13.4 |  |  |  |  |
| pH | 7.80 | 7.85 | 8.31 | 7.63 | 7.81 | 7.94 | 7.61 |  |  |  |  |
| Flow  (m s^-1^) | 1.25 | n/a | 1.20 | 2.60 | 1.40 | 1.20 | 1.30 |  |  |  |  |
| Nitrate  (mg L^-1^) | 7.7 | 6.2 | 9.6 | 3.0 | 7.9 | 7.9 | 6.6 |  |  |  |  |
| DOC  (mg L^-1^) | 2.6 | 2.0 | 2.9 | 6.6 | 4.0 | 1.9 | 1.9 |  |  |  |  |

n/a = measurement not available

*Estimated TWA concentration assuming the Chemcatcher^®^ samplers (CC) were in the time integrative mode for all nine compounds over the 14-day deployment.

Table S7(g) Aqueous concentration and estimated time-weighted average (TWA) concentration measured by the Chemcatcher^®^ (CC) (n = 3) for eight acidic herbicides, together with the river water properties in field trial 1 at site 7 (River Exe at Thorverton gauging station (national grid reference co-ordinates SS93580161)). The concentration of acidic herbicides found in the Chemcatcher^®^ field blanks was below the detection limit of the analytical method. Bold type face shows elevated concentrations

| **Acidic herbicide** | Concentration (ng L^-1^) in spot water samples | | | | | | | CC 1  TWA*  (ng L^-1^) | CC 2  TWA*  (ng L^-1^) | CC 3  TWA*  (ng L^-1^) | Average  TWA*  (ng L^-1^) |
| --- | --- | --- | --- | --- | --- | --- | --- | --- | --- | --- | --- |
|  | Day  0 | Day  2 | Day  5 | Day  7 | Day  9 | Day  12 | Day  14 |  |  |  |  |
| 2,4-D | <7 | <7 | <7 | **20** | <7 | <7 | <7 | <1.12 | <1.12 | <1.12 | <1.12 |
| Dicamba | <7 | <7 | <7 | <7 | <7 | <7 | <7 | 2.11 | <1.62 | 1.95 | 2.03 |
| Dichlorprop | <8 | <8 | <8 | <8 | <8 | <8 | <8 | <0.64 | <0.64 | <0.64 | <0.64 |
| Fluroxypyr | <11 | <11 | <11 | <11 | <11 | <11 | <11 | 2.15 | <0.98 | <0.98 | 2.15 |
| MCPA | <8 | <8 | <8 | **94** | **10** | <8 | <8 | **12.7** | **4.38** | **7.37** | **8.14** |
| MCPB | <9 | <9 | <9 | <9 | <9 | <9 | <9 | <1.15 | <1.15 | <1.15 | <1.15 |
| Mecoprop | **35** | <7 | <7 | <7 | <7 | <7 | **36** | **12.0** | **5.18** | **9.29** | **8.83** |
| Triclopyr | <8 | <8 | <8 | <8 | <8 | <8 | <8 | **4.0** | **1.62** | **2.83** | **2.83** |
|  |  |  |  |  |  |  |  |  |  |  |  |
| **Water properties** |  |  |  |  |  |  |  |  |  |  |  |
| Temp  (°C) | 15.4 | 13.5 | 11.0 | 9.6 | 10.0 | 12.9 | 14.4 |  |  |  |  |
| pH | 8.67 | 8.52 | 7.95 | 7.66 | 7.67 | 7.90 | 8.24 |  |  |  |  |
| Flow  (m s^-1^) | n/a | n/a | n/a | n/a | n/a | n/a | n/a |  |  |  |  |
| Nitrate  (mg L^-1^) | 18.5 | 7.8 | 34.0 | 7.0 | 6.1 | 17.2 | 13.3 |  |  |  |  |
| DOC  (mg L^-1^) | 2.9 | 2.4 | 1.8 | 5.8 | 2.6 | 1.6 | 1.9 |  |  |  |  |

n/a = measurement not available

*Estimated TWA concentration assuming the Chemcatcher^®^ samplers (CC) were in the time integrative mode for all nine compounds over the 14-day deployment.

Table S7(h) Aqueous concentration and estimated time-weighted average (TWA) concentration measured by the Chemcatcher^®^ (CC) (n = 3) for eight acidic herbicides, together with the river water properties in field trial 1 at site 8 (River Exe at Northbridge intake (national grid reference co-ordinates SX93009710)). The concentration of acidic herbicides found in the Chemcatcher^®^ field blanks was below the detection limit of the analytical method. Bold type face shows elevated concentrations

| **Acidic herbicide** | Concentration (ng L^-1^) in spot water samples | | | | | | | CC 1  TWA*  (ng L^-1^) | CC 2  TWA*  (ng L^-1^) | CC 3  TWA*  (ng L^-1^) | Average  TWA*  (ng L^-1^) |
| --- | --- | --- | --- | --- | --- | --- | --- | --- | --- | --- | --- |
|  | Day  0 | Day  2 | Day  5 | Day  7 | Day  9 | Day  12 | Day  14 |  |  |  |  |
| 2,4-D | <7 | <7 | <7 | <7 | <7 | <7 | <7 | <1.12 | <1.12 | <1.12 | <1.12 |
| Dicamba | <7 | <7 | <7 | <7 | <7 | <7 | **12** | **3.08** | **2.76** | **2.44** | **2.76** |
| Dichlorprop | <8 | <8 | <8 | <8 | <8 | <8 | <8 | <0.64 | <0.64 | <0.64 | <0.64 |
| Fluroxypyr | <11 | <11 | <11 | <11 | <11 | <11 | <11 | **2.05** | **1.76** | **1.47** | **1.76** |
| MCPA | <8 | <8 | <8 | **39** | **10** | **9** | <8 | **15.90** | **10.60** | **10.48** | **12.33** |
| MCPB | <9 | <9 | <9 | <9 | <9 | <9 | <9 | <1.15 | <1.15 | <1.15 | <1.15 |
| Mecoprop | **35** | **8** | <7 | <7 | <7 | <7 | **80** | **17.00** | **14.79** | **13.02** | **14.94** |
| Triclopyr | <8 | <8 | <8 | <8 | <8 | <8 | <8 | **6.20** | **4.31** | **3.91** | **4.81** |
|  |  |  |  |  |  |  |  |  |  |  |  |
| **Water properties** |  |  |  |  |  |  |  |  |  |  |  |
| Temp  (°C) | 14.9 | 12.0 | 11.7 | 9.5 | 9.3 | 12.3 | 14.5 |  |  |  |  |
| pH | 8.43 | 8.54 | 7.75 | 7.69 | 7.55 | 7.78 | 8.36 |  |  |  |  |
| Flow  (m s^-1^) | n/a | n/a | n/a | n/a | n/a | n/a | n/a |  |  |  |  |
| Nitrate  (mg L^-1^) | 9.6 | 7.9 | 9.7 | 4.0 | 6.6 | 7.1 | 7.0 |  |  |  |  |
| DOC  (mg L^-1^) | 2.7 | 2.2 | 2.9 | 4.1 | 2.7 | 2.0 | 2.1 |  |  |  |  |

n/a = measurement not available

*Estimated TWA concentration assuming the Chemcatcher^®^ samplers (CC) were in the time integrative mode for all nine compounds over the 14-day deployment.


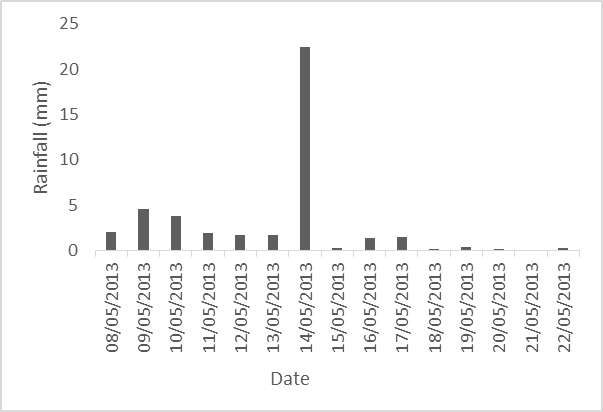


Fig. S6 Rainfall (mm) in the South West England area during the period of field trial 1 (HadUKP - http://www.metoffice.gov.uk/hadobs/hadukp/)

Table S8(a) Aqueous concentration and estimated time-weighted average (TWA) concentration measured by the Chemcatcher^®^ (CC) (n = 2) for eight acidic herbicides, together with the river water properties in field trial 2 at site 1 (River Exe at Ironbridge near Stoodleigh (national grid reference co-ordinates SS94261782)). The concentration of acidic herbicides found in the Chemcatcher^®^ field blanks was below the detection limit of the analytical method. Bold type face shows elevated concentrations

| **Acidic herbicide** | Concentration (ng L^-1^) in spot water samples | | CC 1  TWA*  (ng L^-1^) | CC 2  TWA*  (ng L^-1^) | Average  TWA*  (ng L^-1^) |
| --- | --- | --- | --- | --- | --- |
|  | Day  0 | Day  16 |  |  |  |
| 2,4-D | <7 | <7 | <0.98 | n/a | <0.98 |
| Dicamba | <7 | <7 | <1.42 | n/a | <1.42 |
| Dichlorprop | <8 | <8 | <0.56 | n/a | <0.56 |
| Fluroxypyr | <11 | <11 | <0.86 | n/a | <0.86 |
| MCPA | <8 | <8 | <1.01 | n/a | <1.01 |
| MCPB | <9 | <9 | <1.01 | n/a | <1.01 |
| Mecoprop | <7 | <7 | 1.05 | n/a | 1.05 |
| Triclopyr | **10** | <8 | **4.72** | n/a | **4.72** |
|  |  |  |  |  |  |
| **Water properties** |  |  |  |  |  |
| Temp (°C) | 16.8 | 17.2 |  |  |  |
| pH | 7.84 | 7.93 |  |  |  |
| Flow (m s^-1^) | 0.40 | 0.30 |  |  |  |

n/a = measurement not available

*Estimated TWA concentration assuming the Chemcatcher^®^ samplers (CC) were in the time integrative mode for all nine compounds over the 16-day deployment.

Table S8(b) Aqueous concentration and estimated time-weighted average (TWA) concentration measured by the Chemcatcher^®^ (CC) (n = 2) for eight acidic herbicides, together with the river water properties in field trial 2 at site 2 (Calverleigh Stream at Lower Farleigh (national grid reference co-ordinates SS93111452)). The concentration of acidic herbicides found in the Chemcatcher^®^ field blanks was below the detection limit of the analytical method. Bold type face shows elevated concentrations

| **Acidic herbicide** | Concentration (ng L^-1^) in spot water samples | | CC 1  TWA*  (ng L^-1^) | CC 2  TWA*  (ng L^-1^) | Average  TWA*  (ng L^-1^) |
| --- | --- | --- | --- | --- | --- |
|  | Day  0 | Day  16 |  |  |  |
| 2,4-D | <7 | <7 | <0.98 | <0.98 | <0.98 |
| Dicamba | <7 | <7 | <1.42 | <1.42 | <1.42 |
| Dichlorprop | <8 | <8 | **1.17** | **1.28** | **1.23** |
| Fluroxypyr | **2089** | **27** | **56.85** | **65.67** | **61.26** |
| MCPA | <8 | <8 | **153.33** | **182.86** | **168.09** |
| MCPB | <9 | <9 | <1.01 | <1.01 | <1.01 |
| Mecoprop | <7 | <7 | **2.10** | **2.05** | **2.07** |
| Triclopyr | **5029** | **24** | **137.62** | **157.78** | **147.70** |
|  |  |  |  |  |  |
| **Water properties** |  |  |  |  |  |
| Temp (°C) | 17.8 | 18.2 |  |  |  |
| pH | 7.87 | 7.89 |  |  |  |
| Flow (m s^-1^) | n/a | n/a |  |  |  |

n/a = measurement not available

*Estimated TWA concentration assuming the Chemcatcher^®^ samplers (CC) were in the time integrative mode for all nine compounds over the 16-day deployment.

Table S8(c) Aqueous concentration and estimated time-weighted average (TWA) concentration measured by the Chemcatcher^®^ (CC) (n = 2) for eight acidic herbicides, together with the river water properties in field trial 2 at site 3 (River Lowman at Uplowman pumping station (national grid reference co-ordinates ST00731562)). The concentration of acidic herbicides found in the Chemcatcher^®^ field blanks was below the detection limit of the analytical method. Bold type face shows elevated concentrations

| **Acidic herbicide** | Concentration (ng L^-1^) in spot water samples | | CC 1  TWA*  (ng L^-1^) | CC 2  TWA*  (ng L^-1^) | Average  TWA*  (ng L^-1^) |
| --- | --- | --- | --- | --- | --- |
|  | Day  0 | Day  16 |  |  |  |
| 2,4-D | <7 | <7 | <0.98 | n/a | <0.98 |
| Dicamba | <7 | <7 | <1.42 | n/a | <1.42 |
| Dichlorprop | <8 | <8 | <0.56 | n/a | <0.56 |
| Fluroxypyr | <11 | <11 | <0.86 | n/a | <0.86 |
| MCPA | <8 | <8 | <1.01 | n/a | <1.01 |
| MCPB | <9 | <9 | <1.01 | n/a | <1.01 |
| Mecoprop | <7 | <7 | <0.55 | n/a | <0.55 |
| Triclopyr | <8 | <8 | **20.87** | n/a | **20.87** |
|  |  |  |  |  |  |
| **Water properties** |  |  |  |  |  |
| Temp (°C) | 15.5 | 15.4 |  |  |  |
| pH | 8.09 | 8.14 |  |  |  |
| Flow (m s^-1^) | 0.15 | 0.10 |  |  |  |

n/a = measurement not available

*Estimated TWA concentration assuming the Chemcatcher^®^ samplers (CC) were in the time integrative mode for all nine compounds over the 16-day deployment.

Table S8(d) Aqueous concentration and estimated time-weighted average (TWA) concentration measured by the Chemcatcher^®^ (CC) (n = 2) for eight acidic herbicides, together with the river water properties in field trial 2 at site 4 (Tributary of River Lowman at Uplowman sewage treatment works (national grid reference co-ordinates ST01401542)). The concentration of acidic herbicides found in the Chemcatcher^®^ field blanks was below the detection limit of the analytical method. Bold type face shows elevated concentrations

| **Acidic**  **herbicide** | Concentration (ng L^-1^) in spot water samples | | CC 1  TWA*  (ng L^-1^) | CC 2  TWA*  (ng L^-1^) | Average  TWA*  (ng L^-1^) |
| --- | --- | --- | --- | --- | --- |
|  | Day  0 | Day  16 |  |  |  |
| 2,4-D | <7 | <7 | <0.98 | n/a | <0.98 |
| Dicamba | <7 | <7 | <1.42 | n/a | <1.42 |
| Dichlorprop | <8 | <8 | <0.56 | n/a | <0.56 |
| Fluroxypyr | <11 | <11 | <0.86 | n/a | <0.86 |
| MCPA | <8 | <8 | <1.01 | n/a | <1.01 |
| MCPB | <9 | <9 | <1.01 | n/a | <1.01 |
| Mecoprop | <7 | <7 | <0.55 | n/a | <0.55 |
| Triclopyr | <8 | <8 | <1.18 | n/a | <1.18 |
|  |  |  |  |  |  |
| **Water properties** |  |  |  |  |  |
| Temp (°C) | 14.8 | 15.2 |  |  |  |
| pH | 8.13 | 7.77 |  |  |  |
| Flow (m s^-1^) | n/a | n/a |  |  |  |

n/a = measurement not available

*Estimated TWA concentration assuming the Chemcatcher^®^ samplers (CC) were in the time integrative mode for all nine compounds over the 16-day deployment.

Table S8(e) Aqueous concentration and estimated time-weighted average (TWA) concentration measured by the Chemcatcher^®^ (CC) (n = 2) for eight acidic herbicides, together with the river water properties in field trial 2 at site 5 (River Lowman at confluence with River Exe (national grid reference co-ordinates SS95381200)). The concentration of acidic herbicides found in the Chemcatcher^®^ field blanks was below the detection limit of the analytical method. Bold type face shows elevated concentrations

| **Acidic herbicide** | Concentration (ng L^-1^) in spot water samples | | CC 1  TWA*  (ng L^-1^) | CC 2  TWA*  (ng L^-1^) | Average  TWA*  (ng L^-1^) |
| --- | --- | --- | --- | --- | --- |
|  | Day  0 | Day  16 |  |  |  |
| 2,4-D | <7 | <7 | <0.98 | n/a | <0.98 |
| Dicamba | <7 | <7 | <1.42 | n/a | <1.42 |
| Dichlorprop | <8 | <8 | <0.56 | n/a | <0.56 |
| Fluroxypyr | <11 | <11 | <0.86 | n/a | <0.86 |
| MCPA | <8 | <8 | <1.01 | n/a | <1.01 |
| MCPB | <9 | <9 | <1.01 | n/a | <1.01 |
| Mecoprop | **42** | <7 | **15.87** | n/a | **15.87** |
| Triclopyr | **10** | <8 | **10.85** | n/a | **10.85** |
|  |  |  |  |  |  |
| **Water properties** |  |  |  |  |  |
| Temp (°C) | 15.3 | 15.6 |  |  |  |
| pH | 8.28 | 8.38 |  |  |  |
| Flow (m s^-1^) | 0.20 | 0.10 |  |  |  |

n/a = measurement not available

*Estimated TWA concentration assuming the Chemcatcher^®^ samplers (CC) were in the time integrative mode for all nine compounds over the 16-day deployment.

Table S8(f) Aqueous concentration and estimated time-weighted average (TWA) concentration measured by the Chemcatcher^®^ (CC) (n = 2) for eight acidic herbicides, together with the river water properties in field trial 2 at site 6 (River Dart at Dart Bridge, Bickleigh (national grid reference co-ordinates SS93570762)). The concentration of acidic herbicides found in the Chemcatcher^®^ field blanks was below the detection limit of the analytical method. Bold type face shows elevated concentrations

| **Acidic herbicide** | Concentration (ng L^-1^) in spot water samples | | CC 1  TWA*  (ng L^-1^) | CC 2  TWA*  (ng L^-1^) | Average  TWA*  (ng L^-1^) |
| --- | --- | --- | --- | --- | --- |
|  | Day  0 | Day  16 |  |  |  |
| 2,4-D | <7 | <7 | <0.98 | <0.98 | <0.98 |
| Dicamba | <7 | <7 | <1.42 | <1.42 | <1.42 |
| Dichlorprop | <8 | <8 | <0.56 | <0.56 | <0.56 |
| Fluroxypyr | <11 | <11 | <0.86 | <0.86 | <0.86 |
| MCPA | <8 | <8 | <1.01 | <1.01 | <1.01 |
| MCPB | <9 | <9 | <1.01 | <1.01 | <1.01 |
| Mecoprop | <7 | <7 | **3.76** | **4.37** | **4.07** |
| Triclopyr | <8 | <8 | **6.25** | **7.55** | **6.90** |
|  |  |  |  |  |  |
| **Water properties** |  |  |  |  |  |
| Temp (°C) | 14.7 | 15.3 |  |  |  |
| pH | 8.07 | 7.87 |  |  |  |
| Flow (m s^-1^) | 0.50 | 0.40 |  |  |  |

n/a = measurement not available

*Estimated TWA concentration assuming the Chemcatcher^®^ samplers (CC) were in the time integrative mode for all nine compounds over the 16-day deployment.

Table S8(g) Aqueous concentration and estimated time-weighted average (TWA) concentration measured by the Chemcatcher^®^ (CC) (n = 2) for eight acidic herbicides, together with the river water properties in field trial 2 at site 7 (River Burn at Burn Mill (national grid reference co-ordinates SS94670551)). The concentration of acidic herbicides found in the Chemcatcher^®^ field blanks was below the detection limit of the analytical method. Bold type face shows elevated concentrations

| **Acidic herbicide** | Concentration (ng L^-1^) in spot water samples | | CC 1  TWA*  (ng L^-1^) | CC 2  TWA*  (ng L^-1^) | Average  TWA*  (ng L^-1^) |
| --- | --- | --- | --- | --- | --- |
|  | Day  0 | Day  16 |  |  |  |
| 2,4-D | <7 | <7 | <0.98 | <0.98 | <0.98 |
| Dicamba | <7 | <7 | <1.42 | <1.42 | <1.42 |
| Dichlorprop | <8 | <8 | <0.56 | <0.56 | <0.56 |
| Fluroxypyr | <11 | <11 | **26.63** | **22.95** | **24.79** |
| MCPA | <8 | <8 | <1.01 | <1.01 | <1.01 |
| MCPB | <9 | <9 | <1.01 | <1.01 | <1.01 |
| Mecoprop | <7 | <7 | **2.49** | **2.77** | **2.63** |
| Triclopyr | **14** | <8 | **46.93** | **48.11** | **47.52** |
|  |  |  |  |  |  |
| **Water properties** |  |  |  |  |  |
| Temp (°C) | 14.2 | 15.3 |  |  |  |
| pH | 8.07 | 8.30 |  |  |  |
| Flow (m s^-1^) | 0.30 | 0.20 |  |  |  |

n/a = measurement not available

*Estimated TWA concentration assuming the Chemcatcher^®^ samplers (CC) were in the time integrative mode for all nine compounds over the 16-day deployment.

Table S8(h) Aqueous concentration and estimated time-weighted average (TWA) concentration measured by the Chemcatcher^®^ (CC) (n = 2) for eight acidic herbicides, together with the river water properties in field trial 2 at site 8 (Thorverton Stream at Hulk Lane, Thorverton (national grid reference co-ordinates SS93250158)). The concentration of acidic herbicides found in the Chemcatcher^®^ field blanks was below the detection limit of the analytical method. Bold type face shows elevated concentrations

| **Acidic herbicide** | Concentration (ng L^-1^) in spot water samples | | CC 1  TWA*  (ng L^-1^) | CC 2  TWA*  (ng L^-1^) | Average  TWA*  (ng L^-1^) |
| --- | --- | --- | --- | --- | --- |
|  | Day  0 | Day  16 |  |  |  |
| 2,4-D | <7 | <7 | <0.98 | <0.98 | <0.98 |
| Dicamba | <7 | <7 | <1.42 | <1.42 | <1.42 |
| Dichlorprop | <8 | <8 | <0.56 | <0.56 | <0.56 |
| Fluroxypyr | <11 | <11 | **2.83** | **3.60** | **3.21** |
| MCPA | <8 | <8 | <1.01 | <1.01 | <1.01 |
| MCPB | <9 | **15** | <1.01 | <1.01 | <1.01 |
| Mecoprop | <7 | <7 | **1.71** | **1.71** | **1.71** |
| Triclopyr | **9** | <8 | **13.21** | **15.33** | **14.27** |
|  |  |  |  |  |  |
| **Water properties** |  |  |  |  |  |
| Temp (°C) | 14.4 | 16.0 |  |  |  |
| pH | 7.88 | 8.00 |  |  |  |
| Flow (m s^-1^) | 0.10 | 0.15 |  |  |  |

n/a = measurement not available

*Estimated TWA concentration assuming the Chemcatcher^®^ samplers (CC) were in the time integrative mode for all nine compounds over the 16-day deployment.

Table S8(i) Aqueous concentration and estimated time-weighted average (TWA) concentration measured by the Chemcatcher^®^ (CC) (n = 2) for eight acidic herbicides, together with the river water properties in field trial 2 at site 9 (River Exe at Northbridge intake (national grid reference co-ordinates SX93009710)). The concentration of acidic herbicides found in the Chemcatcher^®^ field blanks was below the detection limit of the analytical method. Bold type face shows elevated concentrations

| **Acidic herbicide** | Concentration (ng L^-1^) in spot water samples | | CC 1  TWA*  (ng L^-1^) | CC 2  TWA*  (ng L^-1^) | Average  TWA*  (ng L^-1^) |
| --- | --- | --- | --- | --- | --- |
|  | Day  0 | Day  16 |  |  |  |
| 2,4-D | <7 | <7 | <0.98 | <0.98 | <0.98 |
| Dicamba | <7 | <7 | <1.42 | <1.42 | <1.42 |
| Dichlorprop | <8 | <8 | **1.51** | **1.62** | **1.56** |
| Fluroxypyr | <11 | <11 | <0.86 | <0.86 | <0.86 |
| MCPA | <8 | <8 | **6.96** | **6.65** | **6.80** |
| MCPB | <9 | <9 | <1.01 | <1.01 | <1.01 |
| Mecoprop | <7 | <7 | **5.31** | **7.19** | **6.25** |
| Triclopyr | <8 | <8 | **10.26** | **14.62** | **12.44** |
|  |  |  |  |  |  |
| **Water properties** |  |  |  |  |  |
| Temp (°C) | 15.5 | 16.9 |  |  |  |
| pH | 7.26 | 7.17 |  |  |  |
| Flow (m s^-1^) | n/a | n/a |  |  |  |

n/a = measurement not available

*Estimated TWA concentration assuming the Chemcatcher^®^ samplers (CC) were in the time integrative mode for all nine compounds over the 16-day deployment.

Table S9 Key conditions used for the gas chromatography-selected ion monitoring mass spectrometry method used for the analysis of acidic pharmaceuticals

| **Condition** | **Value** |
| --- | --- |
| Manufacturer/model | Agilent 6890N/5973N GC-MS |
| Capillary column | J & W fused silica DB-5MS, 30 m x 0.25 mm, 0.25 µm film thickness |
| Carrier gas | Helium (30 cm s^-1^ constant flow mode) |
| Inlet type | Cold on-column (oven track mode) |
| Injection volume | 1 µL |
| Oven temperature programme | 55°C (1 min hold), 15°C min^-1^ to 180°C (no hold), 10°C min^-1^ to 250°C (8.7 min hold) |
| Interface temperature | 280°C |
| Ionisation mode | Electron impact at 70 eV |
| Detection mode | Selected ion monitoring |
| *Acidic pharmaceutical*  Diclofenac  Ibuprofen  Naproxen | Ions monitored (quantification & qualification ion)  214 & 309  161 & 220  185 & 244 |

Table S10 Mean (n = 3) mass (ng) of three acidic pharmaceuticals detected on the 3M Empore™ anion-exchange disk at four sites (see Fig. 1) in River Exe catchment

| **Location in field trial 1** | **Mean mass of acidic pharmaceutical on Chemcatcher^®^ disk (ng)** | | |
| --- | --- | --- | --- |
|  | Diclofenac | Ibuprofen | Naproxen |
| Site 2: River Exe at Exebridge pumping station | <1 | 3 | 4 |
| Site 5: River Exe upstream of Tiverton sewage treatment works | <1 | 7 | 6 |
| Site 6: River Exe downstream of Tiverton sewage treatment works | 2 | 36 | 69 |
| Site 8: River Exe at Northbridge intake | <1 | 15 | 30 |
